# Sugar Influx Sensing by the Phosphotransferase System of *Escherichia coli*

**DOI:** 10.1371/journal.pbio.2000074

**Published:** 2016-08-24

**Authors:** Rahul Somavanshi, Bhaswar Ghosh, Victor Sourjik

**Affiliations:** Max Planck Institute for Terrestrial Microbiology & LOEWE Center for Synthetic Microbiology (SYNMIKRO), Marburg, Germany; Rutgers University-Robert Wood Johnson Medical School, United States

## Abstract

The phosphotransferase system (PTS) plays a pivotal role in the uptake of multiple sugars in *Escherichia coli* and many other bacteria. In the cell, individual sugar-specific PTS branches are interconnected through a series of phosphotransfer reactions, thus creating a global network that not only phosphorylates incoming sugars but also regulates a number of cellular processes. Despite the apparent importance of the PTS network in bacterial physiology, the holistic function of the network in the cell remains unclear. Here we used Förster resonance energy transfer (FRET) to investigate the PTS network in *E*. *coli*, including the dynamics of protein interactions and the processing of different stimuli and their transmission to the chemotaxis pathway. Our results demonstrate that despite the seeming complexity of the cellular PTS network, its core part operates in a strikingly simple way, sensing the overall influx of PTS sugars irrespective of the sugar identity and distributing this information equally through all studied branches of the network. Moreover, it also integrates several other specific metabolic inputs. The integrated output of the PTS network is then transmitted linearly to the chemotaxis pathway, in stark contrast to the amplification of conventional chemotactic stimuli. Finally, we observe that default uptake through the uninduced PTS network correlates well with the quality of the carbon source, apparently representing an optimal regulatory strategy.

## Author Summary

The bacterial phosphotransferase system (PTS) mediates uptake of multiple sugars from the environment and also controls cell physiology and swimming behavior in sugar gradients. In *Escherichia coli* and other bacteria, the PTS consists of a number of sugar-specific branches, interconnected via shared components through a series of phosphotransfer reactions. Whereas most previous studies have focused on understanding individual PTS branches, the holistic function of the entire PTS network in the cell remained elusive. In this study we address this question by investigating the dynamics of multiple protein interactions within the cellular PTS network upon stimulation with sugars and other metabolites. We demonstrate that despite its seeming complexity, the core part of the PTS network operates in a strikingly simple way, sensing the overall influx of PTS sugars and key metabolites into the cell and utilizing this information to control bacterial behavior. We further show that the default influx of the carbon source correlates with its quality, and we use computer simulations to demonstrate that this correlation apparently represents an optimal regulatory strategy.

## Introduction

The phosphotransferase system (PTS) is the major type of bacterial sugar transporters and is present in many bacteria. The PTS mediates uptake of a number of sugars, coupling transport to sugar phosphorylation. The phosphorylation occurs through a series of phosphotransfer reactions, starting from phosphoenolpyruvate (PEP) as a phosphodonor and involving sugar-specific Enzyme II (EII) transporters as well as two general components, Enzyme I (EI) and histidine protein (Hpr) [1,2]. In *Escherichia coli*, EI and Hpr are shared between 15 different sugar-specific branches, which are typically composed of three EII subunits (A, B, and C) that can exist either as a single polypeptide chain or as separate proteins. PTS components are normally phosphorylated in the absence of sugar substrates but become (partly) dephosphorylated in the process of sugar uptake. Consequently, the phosphorylation states of the PTS components serve as an indicator of substrate availability, which is used by bacteria to regulate a number of cellular functions [1,2].

One such regulatory function of the PTS is inducer exclusion, in which the EIIA^Glc^ component of the glucose-specific PTS branch controls uptake of non-PTS sugars lactose and maltose by binding and inhibiting respective transporters [1,3,4]. Uptake of multiple other non-PTS sugars might be controlled by the PTS [1,5–7], but the underlying regulatory mechanisms have not been characterized. While much of the PTS-mediated control of sugar uptake and metabolism was previously thought to occur on the level of gene expression (through the so-called catabolite repression), the importance of the PTS in catabolite repression has been recently questioned [8].

In addition to the control of carbon uptake and metabolism, PTS components are known to have several other regulatory functions. Most prominent among these functions is signaling to the chemotaxis pathway [9–11], which is believed to occur via binding of EI (and likely also EIIA^Glc^) at the cytoplasmic face of the sensory complexes formed by the chemotaxis receptors, the kinase CheA and an adaptor protein CheW [12]. These interactions apparently ensure that the activity of CheA is regulated dependent on the phosphorylation state of EI [13].

Although most studies of the PTS-mediated regulation focused on a single (glucose) branch of the PTS network, other PTS sugars and even non-PTS compounds can elicit similar effects [1,2,14–16]. This indicates a more general regulatory function of the PTS [2], but the processing and integration of these multiple inputs within the network has remained unclear up to now.

Here we used Förster (fluorescence) resonance energy transfer (FRET) to investigate in vivo the dynamic behavior of the PTS network upon stimulation with PTS or non-PTS substrates. We observed that multiple protein interactions within the PTS network are stimulation-dependent, and we used these interactions to investigate the intracellular processing of signals by the PTS network. Our results suggest that the PTS network serves as a global sensor of the sugar influx and also monitors the levels of several non-PTS compounds. This information is integrated and propagated linearly within the network and towards the chemotaxis system, which is in stark contrast to processing of conventional chemotactic stimuli. We further demonstrate strong correlation between the default influx and the quality of the carbon source and propose that such proportional adjustment of the carbon uptake represents an optimal regulatory strategy.

## Results

### Cytoplasmic PTS Components Are Recruited to Transporters upon Stimulation

To understand the cellular dynamics of the PTS network, we tested interactions of over 60 protein pairs using a FRET assay [17,18] (S1 Table). Proteins of interest were C-terminally tagged with cyan and yellow fluorescent proteins (CFP and YFP, respectively) and changes in their pairwise interactions upon stimulation with sugars were monitored using the ratio of the YFP to CFP emission (S1 Fig), which is proportional to the extent of energy transfer and thus to the amount of complex formed by the tagged proteins.

**Fig 1:**
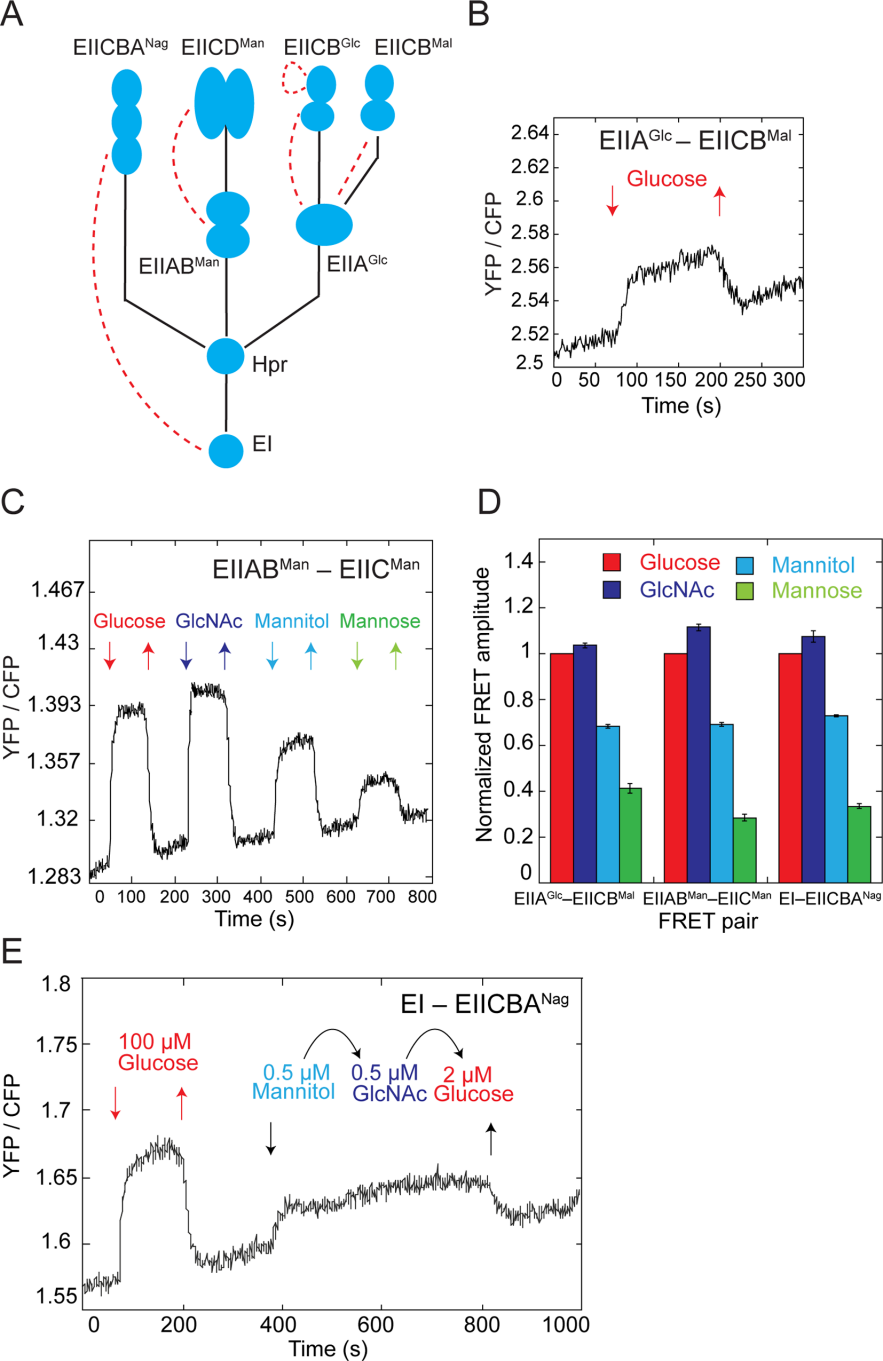
Stimulation dependence of interactions between PTS components. (A) Schematic representation of a part of the PTS network that shows branches for uptake of glucose (EII^Glc^ and EII^Mal^), mannose (EII^Man^), and N-acetylglucosamine (GlcNAc) (EII^Nag^). Phosphotransfer reactions between components are shown with solid lines and observed stimulation-dependent protein interactions are shown with dashed lines. Note that EIIA^Glc^ is shared between EII^Glc^ and EII^Mal^, and EII^Man^ has an additional component EIID^Man^. (B) Example of a FRET measurement for a pair of glucose-specific PTS components. Cells expressing EIIA^Glc^-CFP and EIICB^Mal^-YFP were stimulated by the stepwise addition of PTS sugars and the response was followed as a change in the ratio of YFP to CFP fluorescence. Measurements were acquired every second for a population of several hundred cells, as described in Materials and Methods. (C) Example of a FRET measurement for a EIIAB^Man^-YFP and EIIC^Man^-CFP pair upon stimulation by the stepwise addition of 100 μM of the indicated PTS sugars. (D) The amplitude of FRET response for indicated pairs to saturating levels (100 μM) of PTS sugars, normalized to the glucose response. Error bars indicate standard errors of the mean of three independent replicates. The underlying data for Fig 1D can be found in S1 Data. (E) Signaling equivalency of different PTS sugars. Cells expressing EI-CFP and EIICBA^Nag^ -YFP were stimulated with the indicated subsaturating concentrations of mannitol, GlcNAc or glucose. The response to a saturating glucose stimulus is shown for a reference.

Our analysis revealed nine pairs that were responsive to stimulation by PTS sugars (S1 Table). Among those, four pairs reflected stimulation-dependent changes in interactions between PTS proteins (Fig 1A). These included interactions of EIIA^Glc^ with both of its cognate transporter components that are involved in glucose uptake, EIICB^Mal^ (Fig 1B) and EIICB^Glc^ (S2A Fig), as well as interaction of the cytoplasmic component of the mannose transporter EIIAB^Man^ with its respective membrane component EIIC^Man^ (Fig 1C). Interestingly, in the latter case no interaction of EIIAB^Man^ with another mannose-specific transporter component EIID^Man^ could be detected (S1 Table). Surprisingly, we also observed a stimulation-dependent complex formation between the general PTS component EI and the N-acetylglucosamine (GlcNAc) transporter EIICBA^Nag^ (S2B Fig), which to our knowledge is the first reported interaction of a bacterial EI with sugar-specific PTS components. The observed increase in FRET suggests that in all of these cases cytoplasmic PTS proteins become recruited to the membrane transporters upon exposure to sugars. This increase in interaction was steady, indicating that on the time scale of our experiments, the overall activity of the PTS system shows no adaptation/desensitization in the presence of constant background stimulation.

In addition, we also observed a stimulation-induced reduction in FRET between the two membrane domains (EIIC^Glc^) of the glucose-specific PTS transporter EIICB^Glc^ (S2C Fig). This reduction likely reflects a conformational rearrangement within the transporter upon glucose binding, and it could also be seen in the PTS-deficient strain lacking EI (Δ*ptsI*). No changes in FRET were observed for the other pairs of PTS proteins tested (S1 Table). While most of these pairs are likely to be genuine negatives, in some cases including the expected interaction between EI proteins [19], the efficiency of energy transfer might be too low to be detected by FRET [18].

### PTS Network Senses the Overall Influx of Sugars

We observed that all phosphorylation-dependent interactions between different PTS proteins were similarly responsive to all tested PTS sugars (Figs 1C, S2A and S2B). For example, the increase in the EIIAB^Man^–EIIC^Man^ interaction was stronger upon stimulation with glucose or GlcNAc than with its cognate substrate mannose. Even more strikingly, when compared across different phosphorylation-dependent pairs, relative response amplitudes observed upon stimulation with saturating concentration of different PTS sugars were equal (Fig 1D and S1 Data). This result suggests that EIIA^Glc^, EIIAB^Man^, and EI, and presumably also their membrane interaction partners, are dephosphorylated to the same extent upon stimulation with any PTS sugar. The relative response amplitudes for the EIIA^Glc^–EIICB^Glc^ pair were somewhat different (S2A Fig), indicating additional regulation, but the pair was also responsive to different sugars. These results imply that the phosphorylation state of proteins within the network can rapidly (within seconds) equilibrate, suggesting that reversibility, which has been previously observed for several reactions [20,21], is a general property of the PTS.

Such reversibility of the phosphotransfer reactions means that the PTS network has no intrinsic preference for a specific sugar (e.g., glucose) but rather senses the overall sugar influx into the cell. Indeed, the activity of the EI–EIICBA^Nag^ pair remained unaltered upon exchange of equivalent subsaturating concentrations of mannitol, GlcNAc, or glucose (Fig 1E).

### Inducer Exclusion Is a General Phenomenon

**Fig 2:**
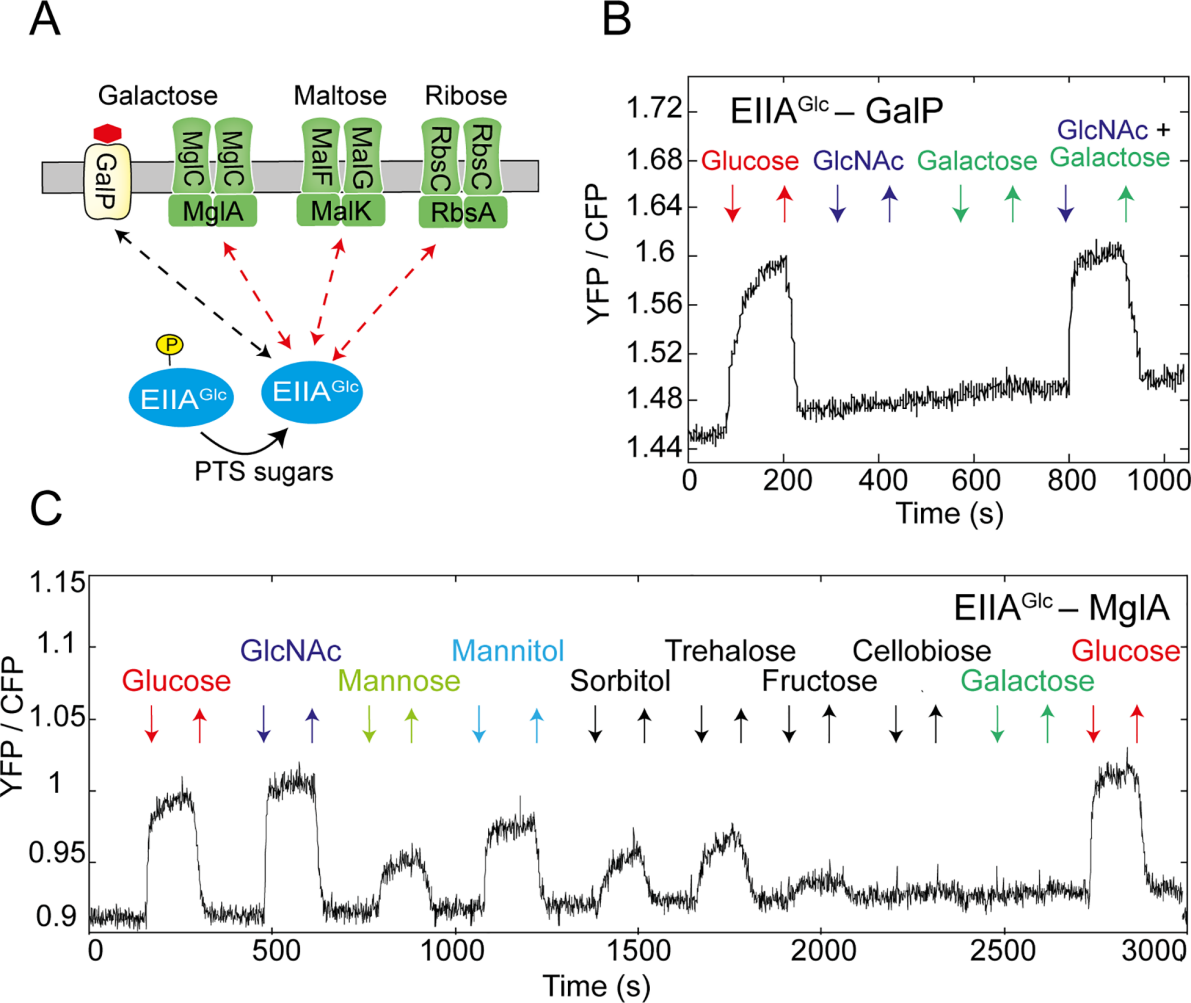
Interactions of EIIA^Glc^ with non-PTS transporters. (A) Summary of the observed interactions between EIIA^Glc^ and non-PTS transporters. (B, C) Measurement of the FRET response to 100 μM of indicated PTS and non-PTS sugars for cells expressing EIIA^Glc^-CFP together with GalP-YFP (B) or MglA-YFP (C).

In addition to the interactions between different PTS proteins, we observed that sugars stimulate interactions of EIIA^Glc^ with a number of transporters for non-PTS sugars (Fig 2A and S1 Table). These include the galactose symporter GalP (Fig 2B) as well as the ATP binding subunits of the maltose, galactose and ribose ABC transporters—MglA, MalK, and RbsA, respectively (Figs 2C, S3A and S3B). Interactions of EIIA^Glc^ with the ABC transporters were stimulated by multiple PTS sugars, apparently directly reflecting the phosphorylation state of EIIA^Glc^. Their response amplitudes were generally consistent with the response within the PTS network (Fig 2A), although additional regulation might exist for the EIIA^Glc^–MalK pair (S3A Fig).

In contrast, the interaction of EIIA^Glc^ with GalP was stimulated only by glucose but not by other PTS sugars, e.g., GlcNAc (Fig 2B). Since glucose (along with galactose) is as a substrate of GalP [22], we hypothesized that this interaction may require not only dephosphorylation of EIIA^Glc^—induced by glucose through the PTS—but also a conformational change induced by glucose binding to GalP. Indeed, an increase in the EIIA^Glc^–GalP interaction could be observed when cells were co-stimulated with GlcNAc and galactose, which combined induce both dephosphorylation of EIIA^Glc^ and the conformational change within GalP.

These interactions likely reflect the inhibition of sugar uptake via inducer exclusion, which was previously only described for the lactose symporter and for the maltose ABC transporter [1,3,4], suggesting that this phenomenon is more general. Although several symporters and ABC transporters of non-PTS sugars showed no stimulation-dependent interactions with EIIA^Glc^ in our FRET assay (S1 Table), some of these interactions might be again too weak to be detected by FRET.

### PTS Signals Propagate Linearly to the Chemotaxis Pathway

**Fig 3:**
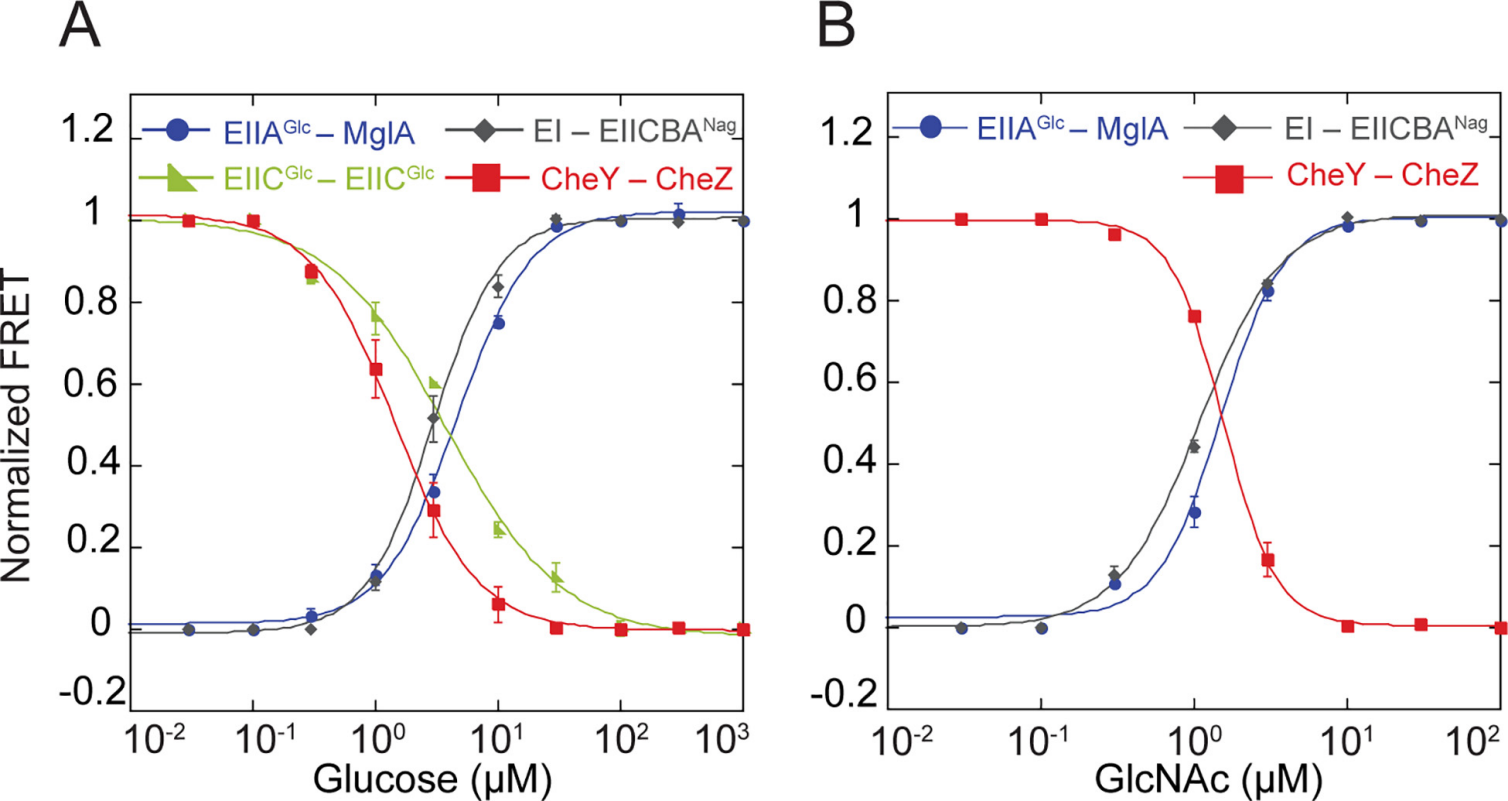
Linear propagation of PTS signals through the network. Dose-response measurements for cells expressing indicated FRET pairs, stimulated by stepwise addition and subsequent removal of varying concentrations of glucose (A) or GlcNAc (B). FRET amplitude for each pair was normalized to the response at saturating stimulation. Data were fitted using a Hill equation (solid lines). Error bars indicate standard error of the mean of three independent experiments. The underlying data for Fig 3A and 3B can be found in S1 Data.

We next investigated the processing of PTS signals and their propagation to the chemotaxis pathway. For that, we measured dose-response curves for two PTS sugars, glucose and GlcNAc using several FRET pairs. These curves showed a clear alignment of response to both sugars (Fig 3A and 3B and S1 Data) at all measured points of the PTS network, including ligand-induced changes in the transporter conformation (for glucose). Nearly identical dose-response curves were also observed for genomic EIIA^Glc^-CFP and MglA-YFP fusions expressed from native promoters (S4 Fig and S1 Data), confirming that no artifacts were generated due to plasmid-based expression of fusion proteins. The observed alignment suggests linear spreading of the PTS signals throughout the network without any amplification or attenuation, consistent with the proportional dephosphorylation of all PTS proteins.

More surprisingly, the dose alignment was also observed at the level of the chemotaxis system. Here we utilized a FRET reporter that relies upon phosphorylation-dependent interaction between the response regulator CheY and its phosphatase CheZ and reflects changes in the activity of the chemotaxis pathway [12,17,23]. Because *E*. *coli* chemotactic response to glucose is known to be mediated by both the chemotaxis receptor (Trg) and by the PTS, we conducted measurements in the strain RS4 that is not capable of chemoreceptor-mediated sensing of glucose. In this background, the CheY–CheZ dose-response curve was consistent with the responses of the PTS FRET pairs (Fig 3A). As positive (attractant) stimuli inhibit the activity of the chemotaxis pathway, this FRET signal decreased with increasing sugar concentration. Similar alignment of the dose-response curves was observed for GlcNAc (Fig 3B), which is sensed solely through the PTS. Furthermore, the dose-response curves for the chemotactic response to glucose and GlcNAc were the same in a receptorless strain expressing only one major chemoreceptor Tar (S5A and S5B Fig and S1 Data), confirming that the PTS-mediated response is independent of the specific set of chemoreceptors [12]. The observed alignment suggests that the PTS signals are not amplified by the chemotaxis system, which is in stark contrast to the processing of signals that originate from ligand binding to the chemoreceptors (see Discussion).

### PTS Integrates and Transmits Additional Metabolic Signals

**Fig 4:**
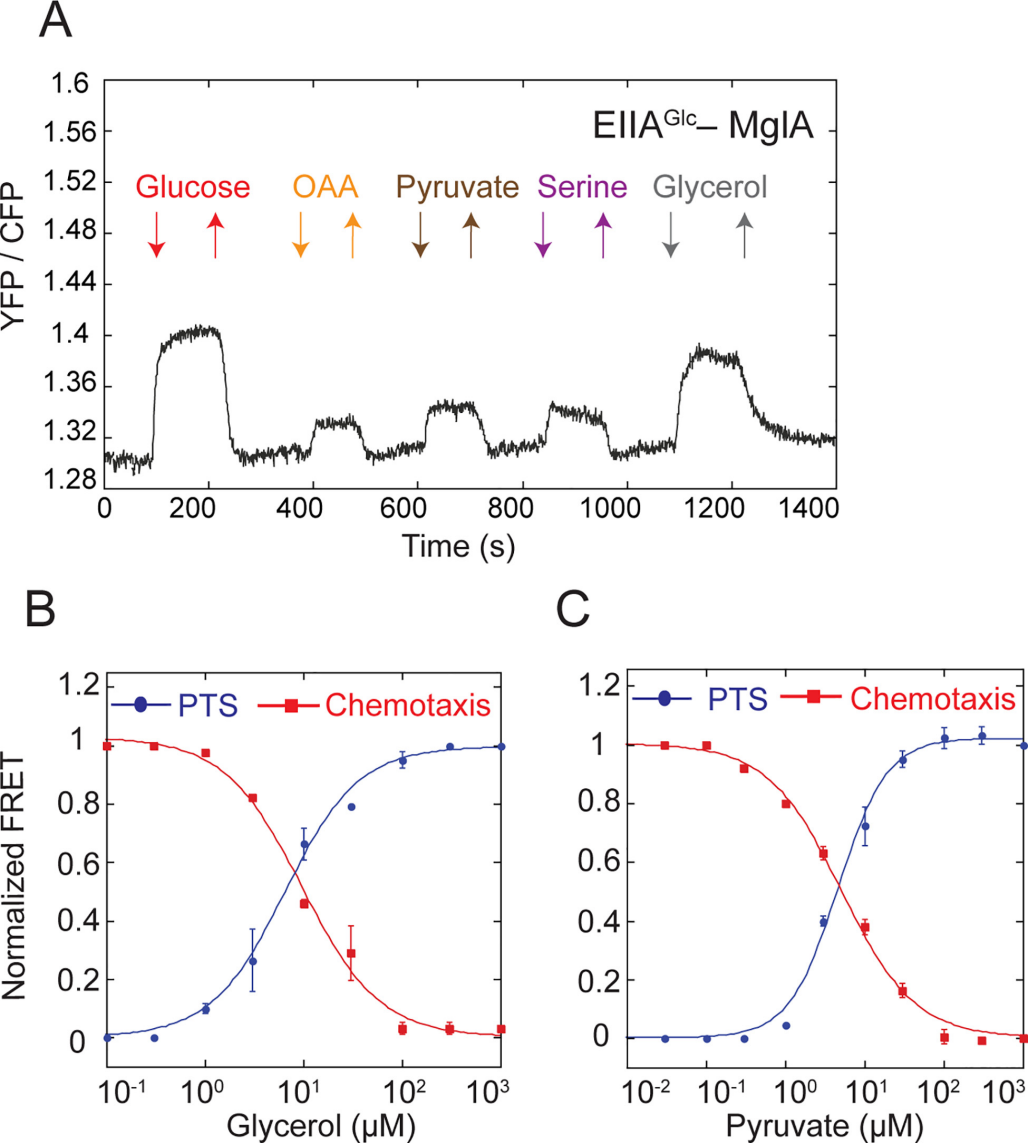
PTS network integrates other metabolic signals. (A) Measurement of the FRET response for cells expressing EIIA^Glc^-CFP and MglA-YFP and stimulated with 100 μM of indicated non-PTS compounds. (B, C) Dose-response measurements for cells expressing EIIA^Glc^-CFP and MglA-YFP (PTS) or CheZ-CFP and CheY-YFP (chemotaxis) pairs and stimulated by stepwise addition and subsequent removal of varying concentrations of glycerol (B) or pyruvate (C). FRET amplitude was normalized to the response at saturating stimulation. Data were fitted using a Hill equation (lines). Error bars indicate standard error of the mean of three independent experiments. The underlying data for Fig 4B and 4C can be found in S1 Data.

Several non-PTS compounds can elicit PTS-like responses, such as catabolite repression, but their connection to the PTS network remained unclear [15]. Moreover, recent work has shown that catabolite repression does not require a functional PTS [8]. To test whether these metabolic signals can nevertheless directly affect the PTS activity, we analyzed the response of several protein pairs upon stimulation with a number of non-PTS metabolites (S2 Table). We observed that glycerol, pyruvate, oxaloacetate, and serine affected protein interactions within the PTS network comparably to the PTS sugars (Fig 4A). These signal were apparently further relayed to the chemotaxis pathway, since the dose-response curves for the EIIA^Glc^–MglA and CheY–CheZ FRET pairs to glycerol and pyruvate aligned in the wild-type cells (Fig 4B and 4C and S1 Data). Both the PTS regulation and the chemotactic response to glycerol were abolished in a strain deleted for the glycerol kinase *glpK* (S6A and S6B Fig), implying that phosphorylation of glycerol is essential for this response.

In contrast to glycerol and pyruvate, the chemotactic responses to serine and oxaloacetate are primarily mediated via the chemoreceptor Tsr. Nevertheless, the PTS apparently provides a secondary sensing system for these compounds, the existence of which in the case of serine has already been postulated [24,25]. This is confirmed by the alignment of the PTS and chemotactic responses in a strain expressing only another major chemoreceptor Tar (S7A and S7B Fig and S1 Data).

Notably, we observed that the PTS responses to saturating concentrations of pyruvate, oxaloacetate and serine were not additive (S8A Fig), suggesting that these compounds affect PTS activity through a shared pathway. In contrast, responses to glycerol and pyruvate were additive, indicating distinct mechanisms of their sensing (S8B Fig).

No PTS response could be observed for a number of other metabolites (S2 Table). Interestingly, these included α-ketoglutarate (S9A and S9B Fig), which has been proposed to coordinate nitrogen and carbon utilization by regulating activity of EI [26]. Furthermore, deletion of the *sucA* gene, which causes excessive intracellular accumulation of α-ketoglutarate [26], had no effect on the response to glucose (S9C Fig).

### Default Uptake of Carbon Sources Correlates with Their Metabolic Efficiency

**Fig 5:**
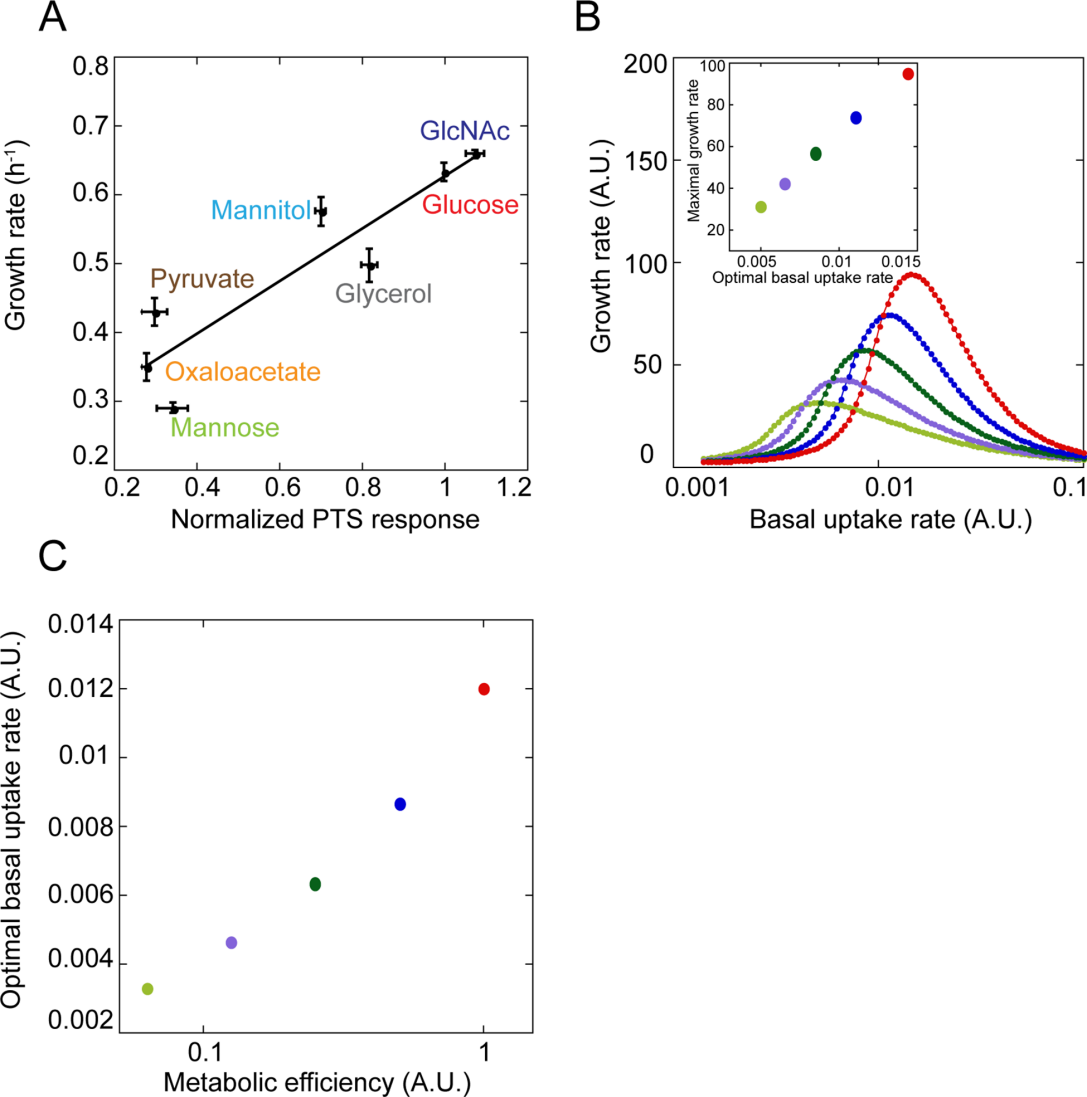
Correlation between default uptake and metabolic efficiency of a carbon source. (A) The averaged relative response of EIIA^Glc^–MglA, EIIAB^Man^–EIIC^Man^ and EI–EIICBA^Nag^ FRET pairs to saturating concentration of indicated metabolites, normalized to the glucose response and plotted against the growth rate of exponential *E*. *coli* culture in minimal medium supplemented with the respective metabolite. Solid line indicates linear fit to the data, with R^2^ = 0.83, *p* = 0.004. The underlying data for Fig 5' A can be found in S1 Data. (B) Simulations of the growth rates at various basal uptake rates, computed for five different values of metabolic efficiency of a carbon source (marked by different colors). Note that the color code is not related to (A). Simulations were performed using the mathematical model described in S1 Text. Inset shows the maximal growth rate as a function of the optimal basal uptake rate for different metabolic efficiency of the carbon source, with the same color code as in the main panel. (C) Correlation between the relative metabolic efficiency of the carbon source and its optimal basal uptake rate for simulated growth on two carbon sources.

Finally, we asked whether the amplitudes of the PTS response to individual metabolites, which reflect their uptake efficiency, could be correlated with the metabolic efficiency of these compounds. Importantly, cells for FRET experiments were grown in a tryptone broth medium, i.e., without sugars, and experiments themselves were performed in the buffer, i.e., in absence of protein expression. Thus, the FRET response reflects the default (basal) sugar uptake by *E*. *coli* cells that had no previous exposure to sugars. In contrast, during growth on most PTS sugars, *E*. *coli* is known to induce transcription of the respective transporters and catabolic enzymes [27–30], thereby adjusting the rates of uptake and catabolism dependent on the carbon source in the medium. Nevertheless, for all tested sugars and other carbon sources that affect activity of the PTS network we observed a strong correlation between the PTS response and the growth rate of *E*. *coli* in a minimal medium supplemented with this carbon source (Fig 5A and S1 Data).

We thus conclude that the default relative uptake efficiency of different carbon sources by non-stimulated *E*. *coli* cells is pre-adjusted to match metabolic efficiency, i.e., the relative growth rates that can be attained on these carbon sources. To test whether such correlation could stem from evolutionary optimization of growth, we mathematically modeled the dependence of the rate of biomass production on the basal rate of uptake (i.e., the rate prior to sugar-specific induction) for sugars with different metabolic efficiency (S1 Text). In our initial model, the transporters are inducible by their phosphorylated substrates, as reported for a number of PTS sugars in *E*. *coli* [29,31,32], and for simplicity we also assumed that maximal fold induction of uptake relative to the basal rate is equal for all sugars. Indeed, we observed that the optimal basal uptake rate increases with the metabolic efficiency of sugar (Fig 5B), and this correlation is roughly linear (Fig 5B Inset), similar to the experimentally observed correlation. Qualitatively, such correlation arises due to a trade-off between energy gained from sugar catabolism and energy spent on sugar uptake, with the higher metabolic efficiency of sugar leading to the higher optimal basal rate of uptake (see S1 Text for details). Importantly, this correlation holds even in the absence of the transporter induction by the phosphorylated sugar (S10A and S10B Fig and S1 Data) or when the maximal induction does not depend on the basal rate of uptake (S10C and S10D Fig and S1 Data). It is thus likely to be also relevant for PTS transporters that rely on different mechanisms of induction, such as those controlled by the PTS regulation domain (PRD)-containing transcriptional regulators [33,34]. Moreover, similar correlation between the optimal basal uptake rate and the metabolic value of the sugar was observed for simulated growth on mixtures of multiple sugars (Figs 5C and S11 and S1 Data).

## Discussion

The bacterial PTS is one of the most prominent examples of a cellular network that combines metabolic and sensory functions. Nevertheless, although the principle mechanism of the PTS-mediated sugar uptake and the sensory functions of its glucose branch are well established [1,2], the holistic operation of the network in the cell—including the interplay between the different branches of the PTS—has up to now remained unknown. In this study, we used an intracellular FRET assay to follow signal processing within the *E*. *coli* PTS network, demonstrating that the network can be seen as a linear sensor of the overall sugar influx into the cell. We observed that multiple protein interactions within the network are similarly affected by every PTS sugar, including alignment of dose-response curves for all measured pairs. Thus, dephosphorylation of any PTS transporter by its substrate must rapidly equilibrate within the network, suggesting that reversibility, which was previously observed for several phosphotransfer reactions [11,20,21,35,36], is a general feature of the PTS network. Here we did not observe any hierarchical preference for the uptake of individual sugars, including glucose, meaning that all sugars compete with each other for uptake. Importantly, such a general view of the network as a global sugar influx sensor does not contradict sugar-specific downstream regulation of cellular functions by individual PTS branches, such as differential dephosphorylation of the transcriptional antiterminator BglG by branch-specific substrates versus other PTS sugars [33,34].

Besides sensing sugar influx, the PTS activity was observed to reflect levels of other metabolites, including pyruvate, glycerol, oxaloacetate, and serine. Sensing of these metabolites might be mediated by the pyruvate to PEP ratio [2,37,38] that influences the phosphorylation state of the PTS network. Notably, pyruvate, oxaloacetate, and serine appear to affect the PTS response through the same pathway, whereas glycerol does not. Nevertheless, a number of other metabolites that were previously implicated in PTS-like responses or proposed to directly regulate PTS, including glucose-6-phosphate, gluconate, lactose, and α-ketoglutarate [26,38,39] did not elicit changes in PTS activity in our experiments.

Another important observation was that the cytoplasmic components of multiple PTS systems become recruited to the respective membrane transporters upon sugar-induced dephosphorylation. These include protein interactions within sugar-specific branches, such as binding of EIIAB^Man^ to EIIC^Man^, but also recruitment of the general PTS component EI to EIICBA^Nag^, both of which have not being reported previously. In the latter case, it is likely that the EI–EIICBA^Nag^ complex may also recruit Hpr, which is expected to transfer the phosphoryl group from EI to EIIA, but that Hpr binding is not sufficiently strong to be detected by FRET. Overall, such formation of transporter complexes could increase the efficiency of phosphotransfer reactions and thus of sugar uptake by relieving diffusional limitation on the reaction rates [1,40–43], although this limitation might be less severe in bacteria than in eukaryotes [44,45]. Interestingly, formation of these transport complexes is stimulated by any PTS sugar and even by the non-PTS compounds, not only by the substrate for the respective PTS transporter.

PTS stimulation also induced interaction of EIIA^Glc^ with several non-PTS sugar transporters, including the ABC transporters for maltose, ribose, and galactose, as well as the galactose symporter (GalP). Thus, inhibition of the non-PTS sugar uptake via inducer exclusion, which has been previously characterized for lactose and maltose, may be a general phenomenon. Notably, whereas dephosphorylation of EIIA^Glc^ is sufficient to induce its interactions with the ABC transporters, its interaction with GalP requires the presence of GalP substrate (galactose or glucose). Such cooperativity in binding of the sugar substrate and dephosphorylated EIIA^Glc^ might represent a general feature of the PTS-mediated regulation of sugar symporters, and possibly also other cellular functions, consistent with previous reports for LacY [1,4,46] and the melibiose symporter MelB [47], as well as for the glycerol kinase [48].

Furthermore, the PTS provides an integrated metabolic input to the chemotaxis system, which reflects not only the overall influx of PTS sugars but also the presence of other metabolites that affect PTS activity. Signaling through the PTS network might thus be at least partly responsible for the metabolism-related tactic responses, summarily known as energy taxis [49,50], as well as for the previously described Tsr-independent response to serine [24,25]. Notably, dose alignment of the chemotactic and the PTS response means that the PTS signals are not further amplified by the chemotaxis system. This stands in contrast to chemoreceptor-mediated signals, which undergo amplification due to allosteric interactions within the chemoreceptor clusters [51–54]. Such lack of amplification implies a fundamental difference between the receptor- and the PTS-mediated chemotactic signaling; for the former, the chemotactic response is observed at concentrations that are significantly lower than ligand binding affinity [55], aiding recognition of the low levels of ligand. In contrast, the PTS-mediated response directly reflects the overall influx of sugars and several other key metabolites, likely enabling cells to maximize the uptake of nutrients by navigating in their environment. Furthermore, the receptor- and the PTS-mediated stimuli apparently induce similar conformational changes in the cytoplasmic part of the chemoreceptors [12]. The observed lack of amplification of PTS signals thus indicates that chemoreceptor-mediated signals might be amplified through interactions among membrane-proximal parts of the receptors, in an apparent contradiction to a recently proposed model [56] of signal amplification through interactions between CheW and CheA at the cytoplasmic face of the receptor clusters.

Finally, we observed a strong correlation between the PTS response to saturating levels of individual metabolites and the growth rate on these metabolites as a carbon source. This correlation is highly nontrivial considering that the PTS activity measurements were performed on non-growing cells that had no prior exposure to sugars and thus reflect the basal (default) rate of sugar uptake in uninduced cells. In contrast, during growth *E*. *coli* is free to regulate gene expression to maximize sugar uptake and catabolism, and this induced uptake rate does not need to reflect the default rate. Nevertheless, default uptake rates are apparently pre-adjusted in *E*. *coli* to reflect the metabolic efficiency of the substrate, i.e., growth rates that are achieved on a particular carbon source. Using computer simulations, we could show that such tuning of the default uptake enables *E*. *coli* to maximize its growth rate upon exposure to different sugars, apparently representing an optimal regulation strategy.

## Materials and Methods

### Plasmids and Strains

Plasmids and strains used in this study are listed in S3 Table. *E*. *coli* LJ 110 strain [57] and its derivatives were used in all experiments. All fluorescent protein fusions were constructed by fusing CFP or YFP to the C-terminal end of proteins and were cloned in pTrc99a or pBAD33 expression plasmids, inducible by IPTG [58] or arabinose [59], respectively. Functionality of the fluorescent protein fusions to the core components of the PTS has been shown previously [12]. Strain RS1 that carries chromosomal *crr-cfp* and *mglA-yfp* fusions was constructed by the gene gorging method as described previously [60] and using modified donor vector pKD13 [61].

### Cell Growth and Preparation for FRET Experiments

Cells were grown in tryptone broth (TB 1% and 0.5% NaCl) with antibiotics (100 μg/ml ampicillin, 50 μg/ml kanamycin, 34 μg/ml chloramphenicol) and inducers added at appropriate concentrations (S3 Table). Overnight cultures were grown at 30°C, diluted 1:100, and grown at 34°C and 275 rpm on a rotary shaker. Cells were harvested at OD_600_ of 0.5–0.6 by centrifugation (10,000 rpm, 5 min) and washed thrice with tethering buffer (10 mM KPO4, 0.1 mM EDTA, 1 μM methionine, 10 mM lactic acid, pH 7) that is typically used for FRET experiments [17,18], and resuspended in 2 ml of tethering buffer. Cells were stored at 4°C prior to FRET measurements.

### Growth Rate Measurements on Different Carbon Sources

*E*. *coli* LJ110 was grown in M9 minimal medium (40 mM Na_2_HPO_4_, 22 mM KH_2_PO_4_, 8.5 mM NaCl, 18 mM NH_4_Cl, 0.5 mM CaCl_2_, 10 mM MgSO_4_) supplemented with 0.4% carbon source and 0.2% casamino acids at 34°C and 275 rpm on a rotary shaker. Growth rate was calculated by measurements of OD_600_ during the exponential phase.

### FRET Measurements

FRET measurements were performed as described previously [17,18] on a custom-modified Zeiss Axiovert 200 microscope. Cells were concentrated and attached to poly-L-lysine coated coverslips and placed into a flow chamber of 50 μl volume, which was kept under constant flow (300 μl/min) of tethering buffer by a syringe pump (Harvard Apparatus). Cells were stimulated with sugar solutions in tethering buffer by rapid exchange of buffer reservoir. CFP fluorescence was excited at 436/20 nm through a 455 nm dichroic mirror by a 75 W Xenon lamp. To detect CFP and YFP emissions, 480/40 nm band pass and 520 nm long pass emission filters were used, respectively. The signal was collected with integration time of 1 s using Peltier-cooled photon counters (Hamamatsu) equipped with a PCI-6034 counting board connected to a computer with custom written LabView7 software (National Instruments). FRET amplitude was calculated from changes in ratio of yellow and cyan fluorescence signals. Acquired data was fitted to multisite Hill model for calculation of EC_50_ as described before [17].

### Mathematical Model of PTS-Mediated Regulation

Function of the PTS was modeled using a set of ordinary differential equations (ODEs; see S1 Text). The model assumes that sugar is converted to sugar-phosphate during uptake in a two-step process, and that sugar-phosphate is subsequently converted to ATP (energy). The rate of ATP production is assumed to depend on the metabolic efficiency of the sugar. Fractions of ATP are further utilized for producing the biomass as well as for sugar uptake and for expression of the metabolic enzymes. The final biomass produced within a certain amount of time is assumed to reflect the growth rate. The model further includes transcriptional induction of the sugar-specific transporter and metabolic enzymes by sugar-phosphate. The ODEs were constructed based on mass action kinetics. Simulations of this model were performed by varying the basal uptake rate and the metabolic efficiency, either for growth on single sugar or on a mixture of multiple sugars. The basal uptake rate for which the growth rate is maximal was defined as the optimal basal uptake rate for a particular sugar. Moreover, we derived a simple analytical equation that illustrates the relation between the optimal uptake rate and the metabolic efficiency of the sugar.

## Supporting Information

S1 Fig FRET measurements of stimulation dependence of protein interactions. (A) Setup used for FRET measurements of stimulation dependence of protein-protein interactions, adapted from [1]. See text for details. (B) Cartoon demonstrating stimulation-dependent FRET between EIIA^Glc^-CFP and MglA-YFP. (C) Corresponding FRET measurement upon stimulation with stepwise addition of 100 μM of PTS (glucose) or non-PTS (galactose) sugar. FRET response was followed as change in the ratio of YFP to CFP fluroscence. Measurements were acquired every second as described in Materials and Methods. Image credit: Kentner D, Sourjik V. Dynamic map of protein interactions in the *Escherichia coli* chemotaxis pathway. *Mol Syst Biol*. 2009;5:238. 10.1038/msb.2008.77. 19156130; PubMed Central PMCID: PMCPMC2644175.
(TIF)

S2 Fig Stimulation-dependent interactions between PTS components. Cells expressing EIIA^Glc^-CFP and EIICB^Glc^-YFP (A), EI-CFP and EIICBA^Nag^-YFP (B) and EIIC^Glc^-CFP and EIIC^Glc^-YFP (C) were stimulated with stepwise addition of 100 μM of indicated PTS sugars in a flow chamber and response was monitored as described in Fig 1 and S1 Fig.
(TIF)

S3 Fig Interactions between EIIA^Glc^ and ABC transporters. FRET measurements for cells expressing EIIA^Glc^-CFP with either MalK-YFP (A) or RbsA-YFP (B) that were stimulated with 100 μM of indicated PTS sugars.
(TIF)

S4 Fig FRET measurements using genomic and plasmid-expressed protein fusions. (A) Dose-response curves of FRET measurements for cells expressing genomic fluorescent protein fusions, EIIA^Glc^-CFP and MglA-YFP, from native promoters. For comparison, measurements using plasmid-expressed fusions, taken from Fig 3A, are shown. Data were fitted using a Hill equation (lines). Error bars indicate standard error of the mean of three independent experiments. The underlying data for Panel A can be found in S1 Data. (B) Fluorescence images of genomic fluorescent protein fusions, EIIA^Glc^-CFP and MglA-YFP.
(TIF)

S5 Fig Dose-response measurements for cells expressing only Tar chemoreceptors. Dose responses to glucose (A) or GlcNAc (B) were measured using CheY-YFP and CheZ-CFP FRET pair in cells expressing only Tar. For comparison, dose responses measured using PTS FRET pair (EIIA^Glc^-CFP and MglA-YFP) from Fig 3 are shown. Data were fitted using a Hill equation (lines). Error bars indicate standard error of the mean of three independent experiments. The underlying data can be found in S1 Data.
(TIF)

S6 Fig PTS and chemotaxis response to glycerol in *glpK* deletion strain. Δ*glpK* cells expressing either EIIA^Glc^-CFP and MglA-YFP (A) or CheZ-CFP and CheY-YFP (B) FRET pairs were stimulated with indicated concentration of glucose or glycerol. As a positive control for the chemotaxis response, cells were stimulated with a chemoreceptor-specific attractant, α-methyl-DL-aspartate (MeAsp).
(TIF)

S7 Fig PTS and chemotaxis response to serine and oxaloacetate in Tar-only strain. PTS and chemotaxis response to stepwise addition and subsequent removal of varying concentrations of serine (A) and oxaloacetate (B), measured as in Fig 4 using EIIA^Glc^-CFP and MglA-YFP (PTS) or CheZ-CFP and CheY-YFP (chemotaxis) FRET pairs, respectively. Chemotactic response was measured in cells expressing only Tar from a plasmid. Data were fitted using a Hill equation (lines). Error bars indicate standard error of the mean of three independent experiments. The underlying data can be found in S1 Data.
(TIF)

S8 Fig Processing and integration of non-PTS signals by the PTS network. (A, B) Cells expressing EIIA^Glc^-CFP and MglA-YFP were stimulated with indicated concentrations of non-PTS compounds either alone or in combination.
(TIF)

S9 Fig α-Ketoglutarate has no effect on PTS activity. (A, B) Cells expressing EIIA^Glc^-CFP and MglA-YFP (A) or EI-CFP and EIICBA^Nag^-YFP (B) FRET pairs were stimulated with indicated concentration of α-ketoglutarate and glucose either alone or in combination. (C) Cells carrying *sucA* deletion and expressing EIIA^Glc^-CFP and MglA-YFP FRET pair were stimulated with indicated concentration of glucose individually and in combination with α-ketoglutarate.
(TIF)

S10 Fig Correlation between uptake rate and metabolic efficiency of a carbon source in absence of transporter induction or at fixed induction. (A, B) Dependence of simulated cell growth on uptake rate for carbon sources of different metabolic efficiency for simulations performed as in Fig 5B but in absence of transporter induction by sugar-phosphate (A), and corresponding correlation between the optimal uptake rate and maximal growth rate for carbon sources of different metabolic efficiency (B). (C, D) Same as above but simulated for fixed maximal induction of transporter by sugar phosphate. See S1 Text for details. The underlying data can be found in S1 Data.
(TIF)

S11 Fig Optimal basal uptake rates for a mixture of three sugars. Correlation between the relative metabolic efficiency of the carbon source and its optimal basal uptake rate for simulated growth on three carbon sources. The underlying data can be found in S1 Data.
(TIF)

S1 Table FRET mapping of stimulus-dependent interactions between PTS proteins. (DOCX)

S2 Table List of metabolites tested for PTS response. (DOCX)

S3 Table Lists of plasmids and strains used this study. (DOCX)

S1 Text Mathematical model of PTS-mediated regulation. (PDF)

S1 Data Underlying data for Figs 1D, 3A, 3B, 4B, 4C, 5A, and S4A, S5A, S5B, S7A, S7B, S10A, S10B, S10C, S10D, S11 Figs. (XLSX)

## Abbreviations

CFP: cyan fluorescent protein
EI: Enzyme I
EII: Enzyme II
FRET: Förster resonance energy transfer
Hpr: histidine protein
PEP: phosphoenolpyruvate
PRD: PTS regulation domain
PTS: phosphotransferase system
YFP: yellow fluorescent protein
