# Supplementary figures and images for: Sugar Influx Sensing by the Phosphotransferase System of Escherichia coli
Source: PLoS Biol. 2016 Aug 24;14(8):e2000074. doi: 10.1371/journal.pbio.2000074 (PMC4996493; doi:10.1371/journal.pbio.2000074)

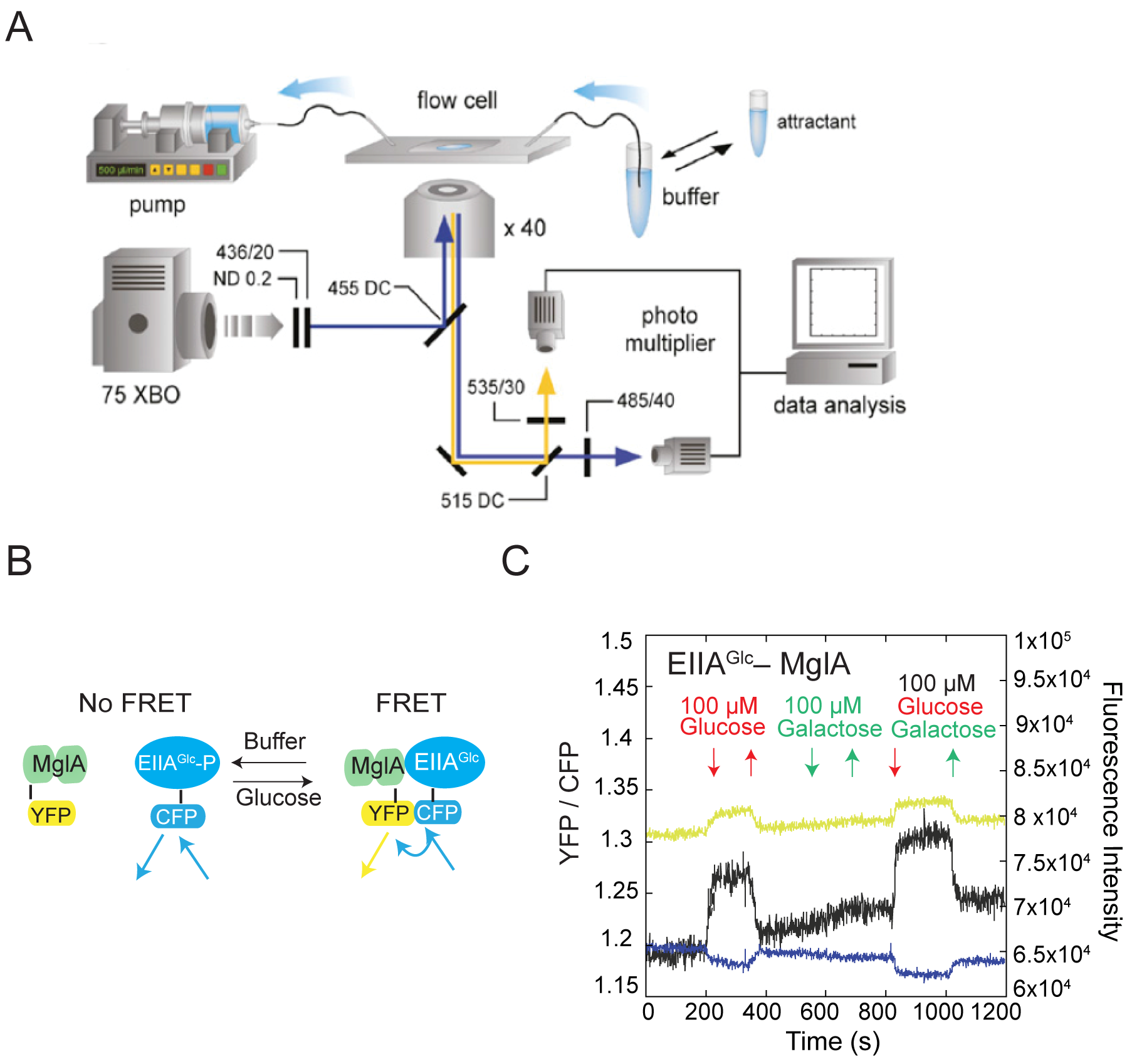

Supplement: S1 Fig — (A) Setup used for FRET measurements of stimulation dependence of protein-protein interactions, adapted from [1]. See text for details. (B) Cartoon demonstrating stimulation-dependent FRET between EIIAGlc-CFP and MglA-YFP. (C) Corresponding FRET measurement upon stimulation with stepwise addition of 100 μM of PTS (glucose) or non-PTS (galactose) sugar. FRET response was followed as change in the ratio of YFP to CFP fluroscence. Measurements were acquired every second as described in Materials and Methods. Image credit: Kentner D, Sourjik V. Dynamic map of protein interactions in the Escherichia coli chemotaxis pathway. Mol Syst Biol. 2009;5:238. 10.1038/msb.2008.77. 19156130; PubMed Central PMCID: PMCPMC2644175. (TIF) [file pbio.2000074.s001.tif]

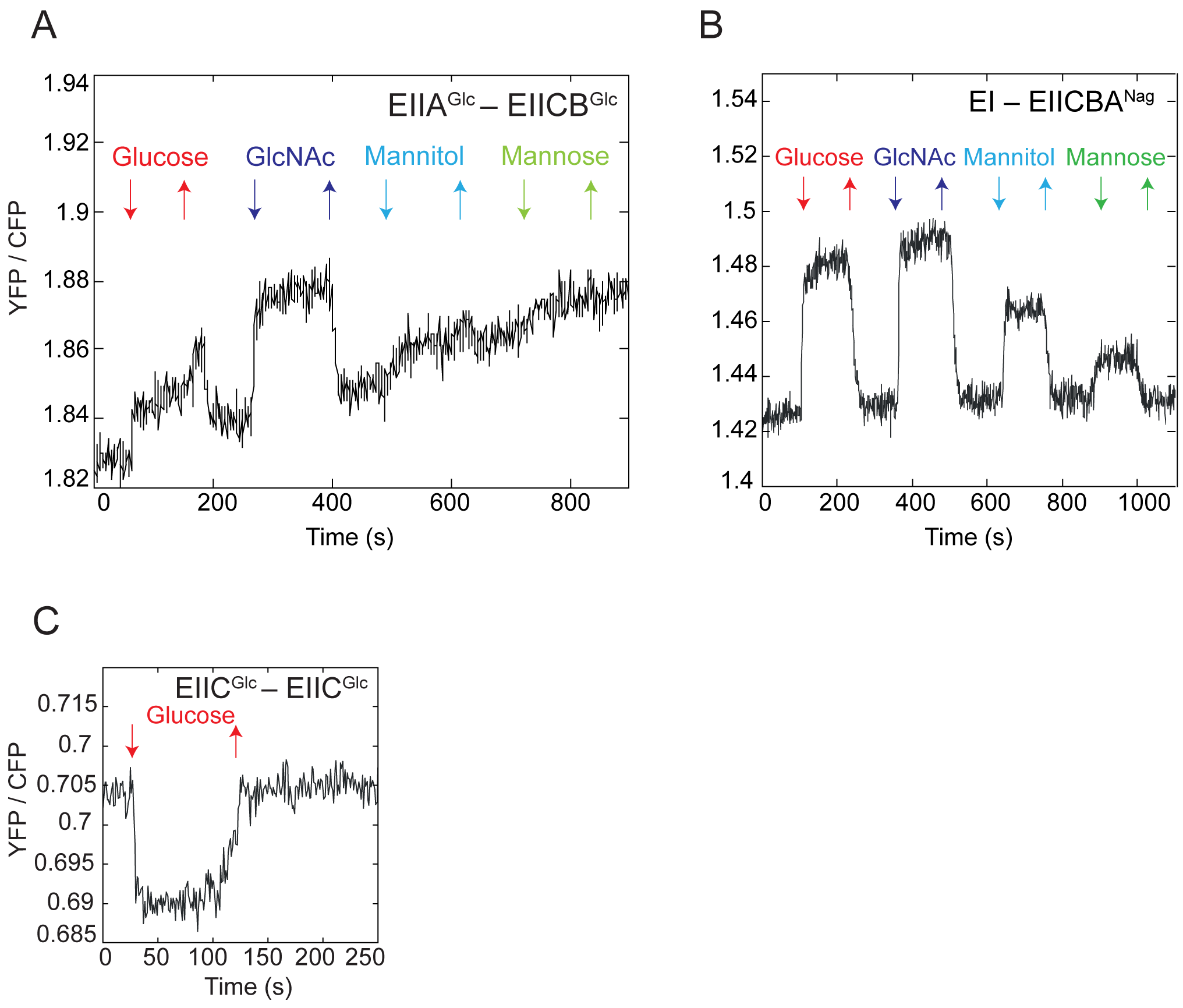

Supplement: S2 Fig — Cells expressing EIIAGlc-CFP and EIICBGlc-YFP (A), EI-CFP and EIICBANag-YFP (B) and EIICGlc-CFP and EIICGlc-YFP (C) were stimulated with stepwise addition of 100 μM of indicated PTS sugars in a flow chamber and response was monitored as described in Fig 1 and S1 Fig. (TIF) [file pbio.2000074.s002.tif]

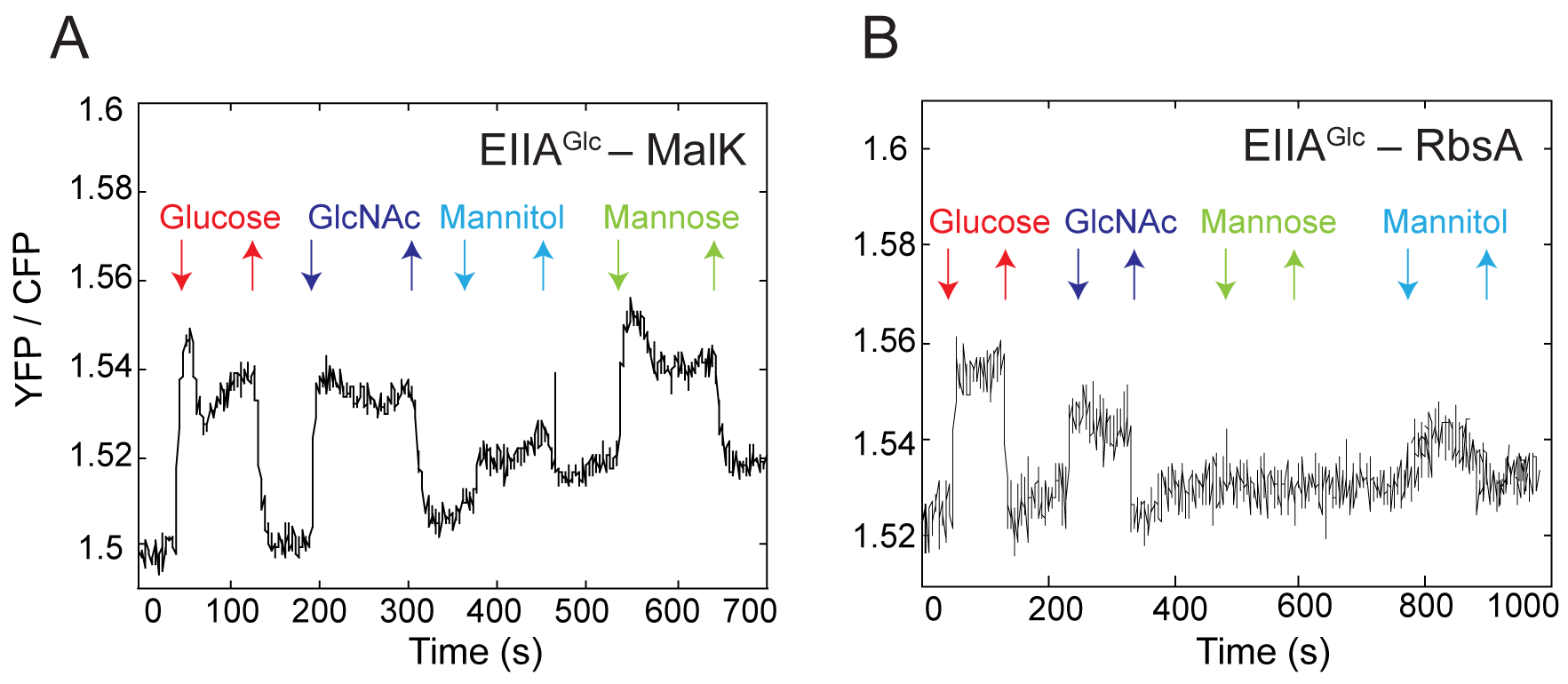

Supplement: S3 Fig — FRET measurements for cells expressing EIIAGlc-CFP with either MalK-YFP (A) or RbsA-YFP (B) that were stimulated with 100 μM of indicated PTS sugars. (TIF) [file pbio.2000074.s003.tif]

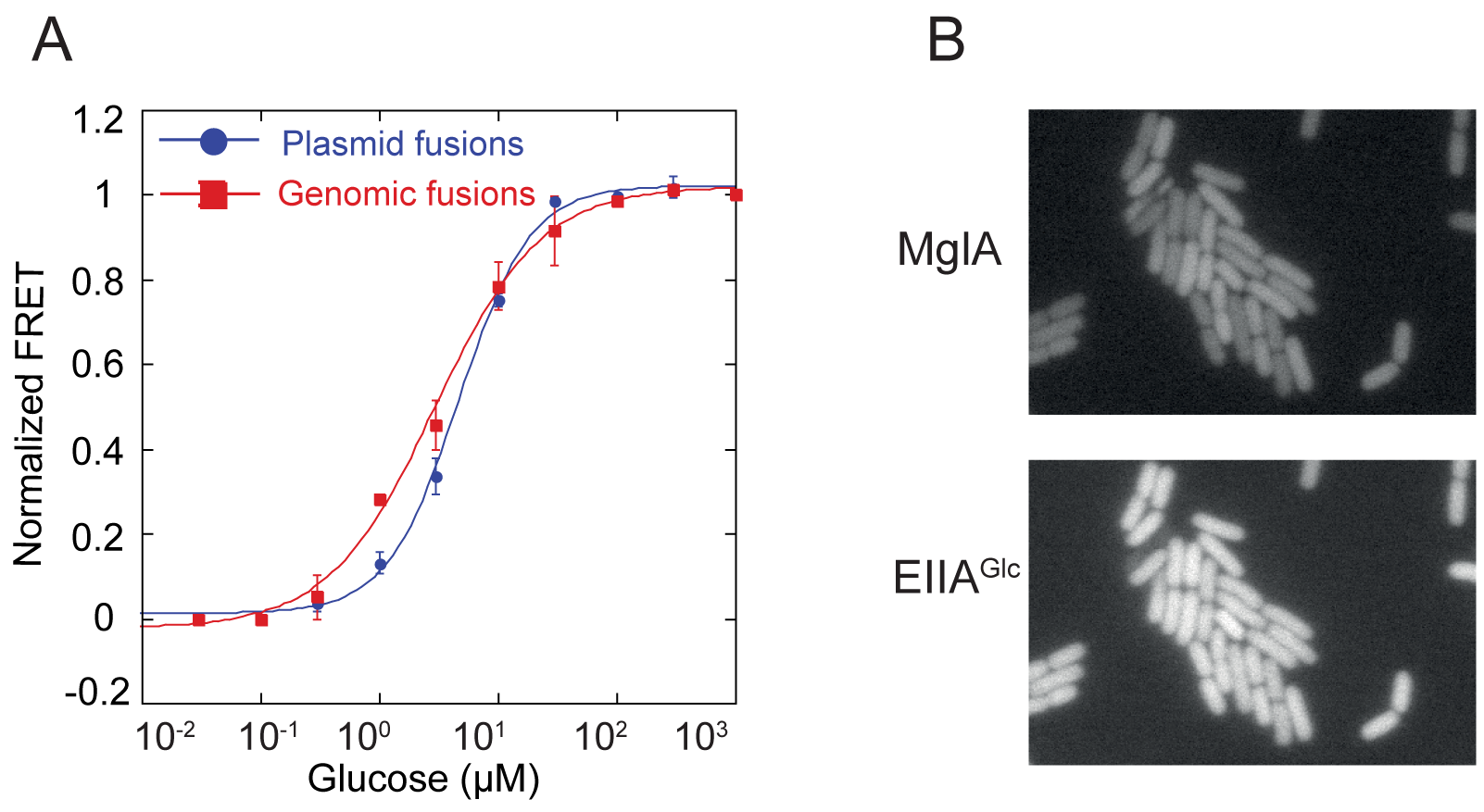

Supplement: S4 Fig — (A) Dose-response curves of FRET measurements for cells expressing genomic fluorescent protein fusions, EIIAGlc-CFP and MglA-YFP, from native promoters. For comparison, measurements using plasmid-expressed fusions, taken from Fig 3A, are shown. Data were fitted using a Hill equation (lines). Error bars indicate standard error of the mean of three independent experiments. The underlying data for Panel A can be found in S1 Data. (B) Fluorescence images of genomic fluorescent protein fusions, EIIAGlc-CFP and MglA-YFP. (TIF) [file pbio.2000074.s004.tif]

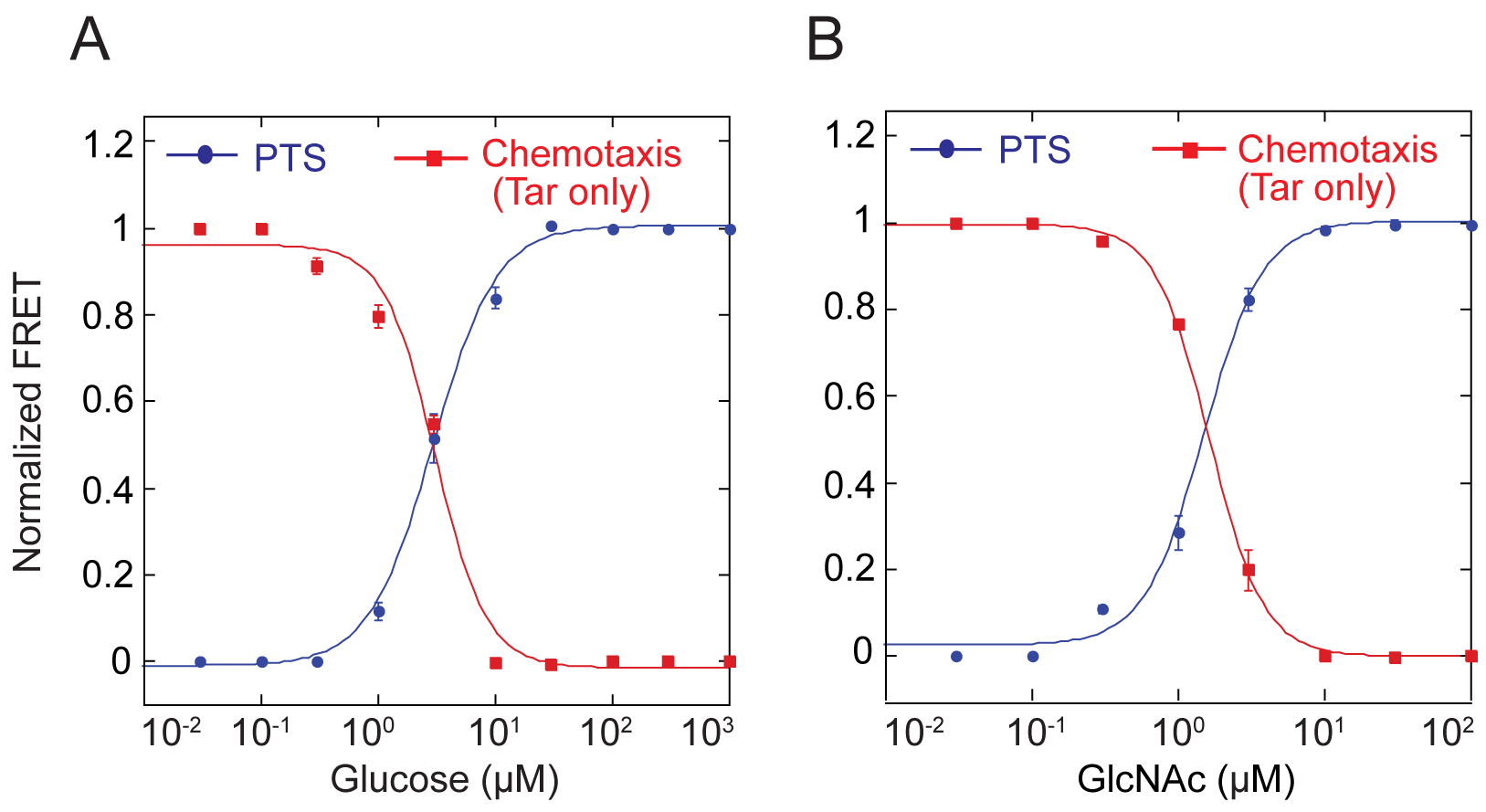

Supplement: S5 Fig — Dose responses to glucose (A) or GlcNAc (B) were measured using CheY-YFP and CheZ-CFP FRET pair in cells expressing only Tar. For comparison, dose responses measured using PTS FRET pair (EIIAGlc-CFP and MglA-YFP) from Fig 3 are shown. Data were fitted using a Hill equation (lines). Error bars indicate standard error of the mean of three independent experiments. The underlying data can be found in S1 Data. (TIF) [file pbio.2000074.s005.tif]

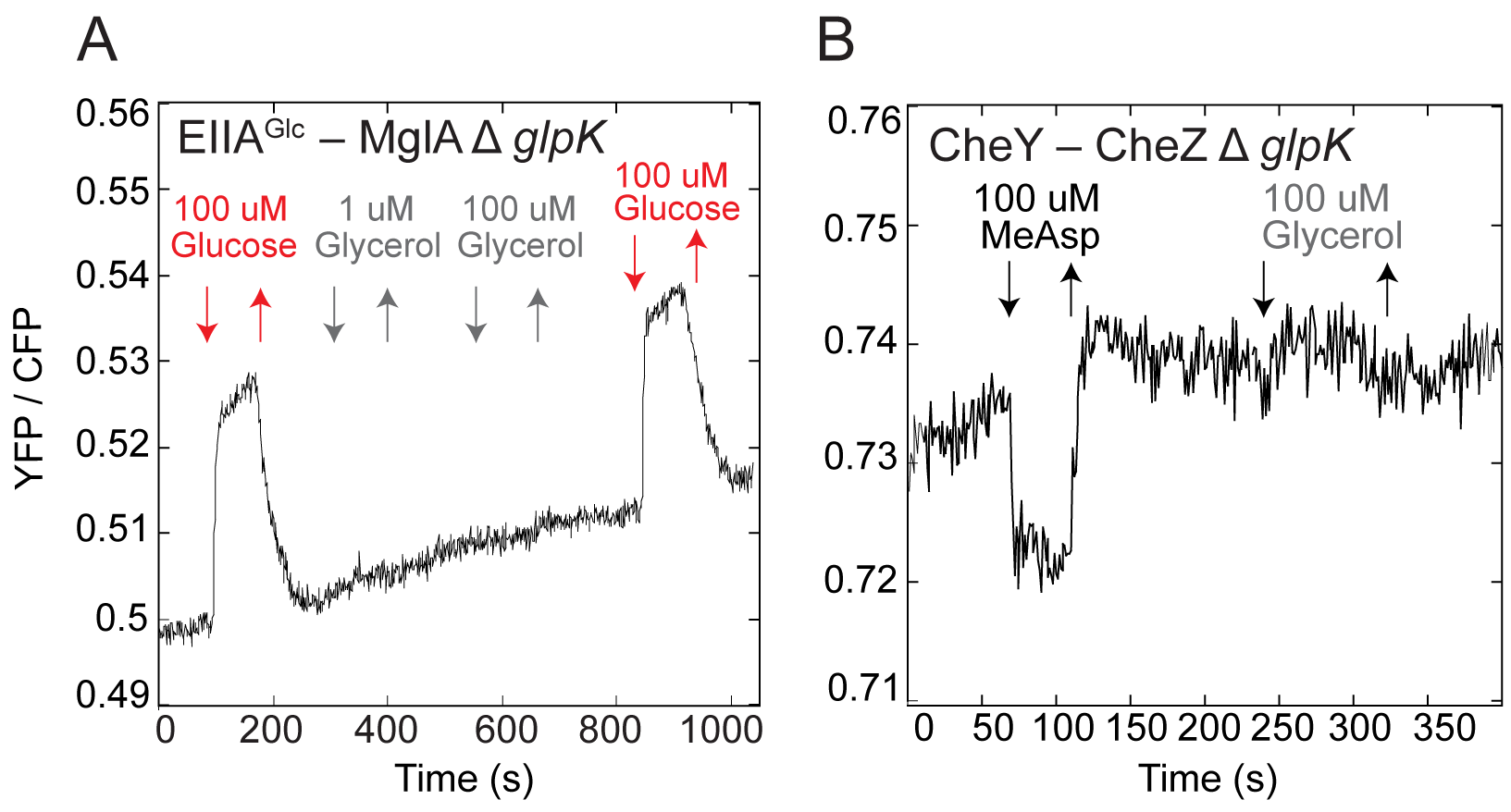

Supplement: S6 Fig — ΔglpK cells expressing either EIIAGlc-CFP and MglA-YFP (A) or CheZ-CFP and CheY-YFP (B) FRET pairs were stimulated with indicated concentration of glucose or glycerol. As a positive control for the chemotaxis response, cells were stimulated with a chemoreceptor-specific attractant, α-methyl-DL-aspartate (MeAsp). (TIF) [file pbio.2000074.s006.tif]

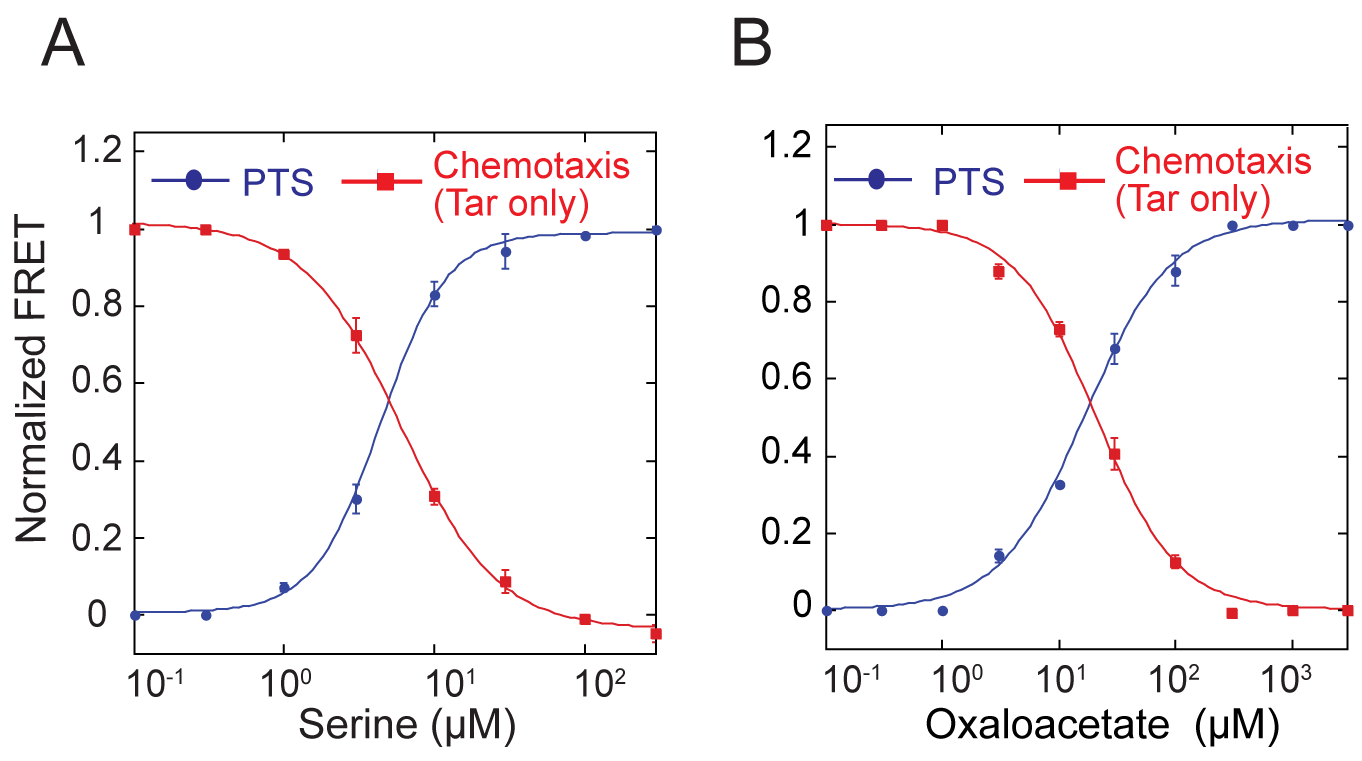

Supplement: S7 Fig — PTS and chemotaxis response to stepwise addition and subsequent removal of varying concentrations of serine (A) and oxaloacetate (B), measured as in Fig 4 using EIIAGlc-CFP and MglA-YFP (PTS) or CheZ-CFP and CheY-YFP (chemotaxis) FRET pairs, respectively. Chemotactic response was measured in cells expressing only Tar from a plasmid. Data were fitted using a Hill equation (lines). Error bars indicate standard error of the mean of three independent experiments. The underlying data can be found in S1 Data. (TIF) [file pbio.2000074.s007.tif]

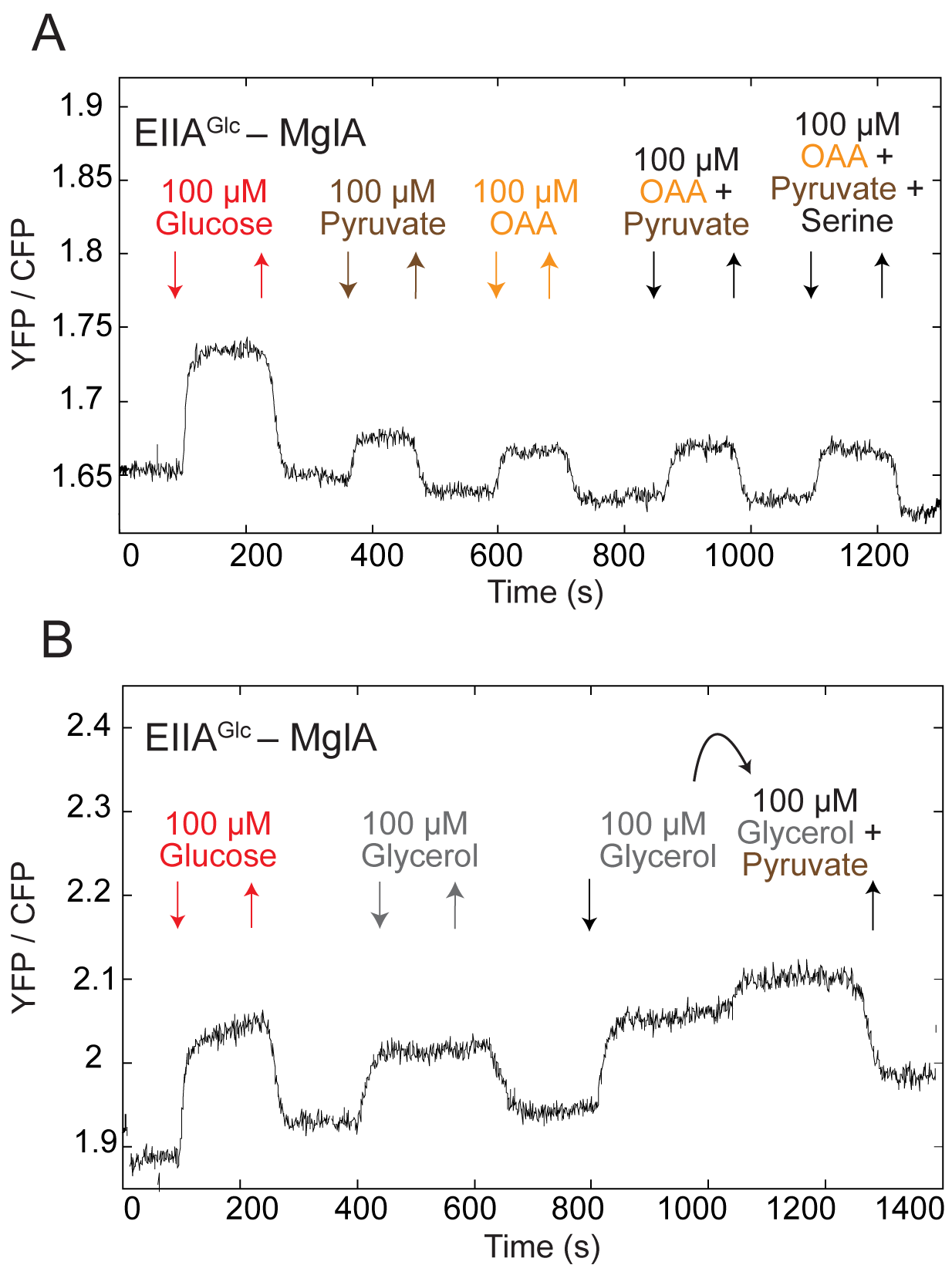

Supplement: S8 Fig — (A, B) Cells expressing EIIAGlc-CFP and MglA-YFP were stimulated with indicated concentrations of non-PTS compounds either alone or in combination. (TIF) [file pbio.2000074.s008.tif]

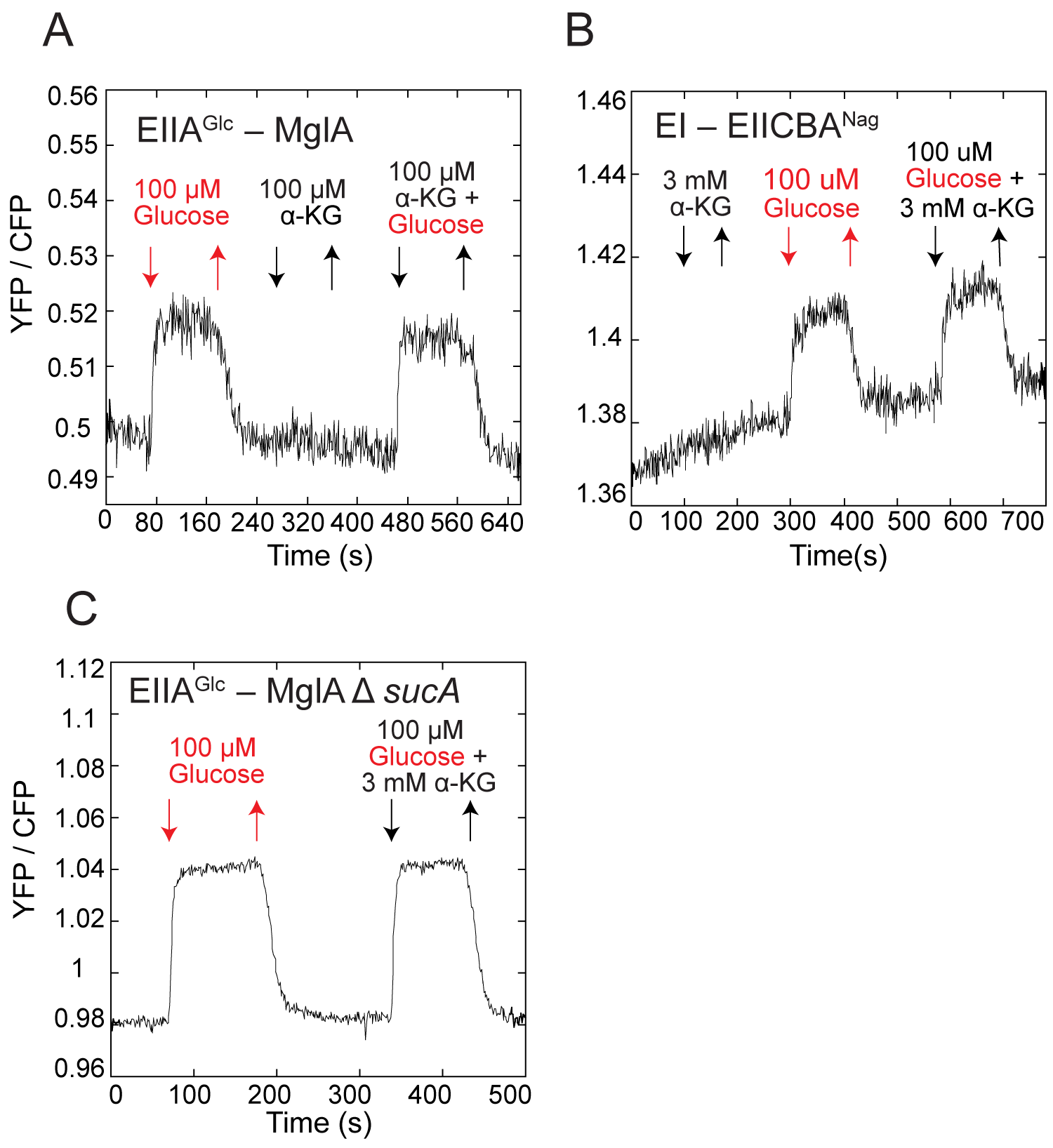

Supplement: S9 Fig — (A, B) Cells expressing EIIAGlc-CFP and MglA-YFP (A) or EI-CFP and EIICBANag-YFP (B) FRET pairs were stimulated with indicated concentration of α-ketoglutarate and glucose either alone or in combination. (C) Cells carrying sucA deletion and expressing EIIAGlc-CFP and MglA-YFP FRET pair were stimulated with indicated concentration of glucose individually and in combination with α-ketoglutarate. (TIF) [file pbio.2000074.s009.tif]

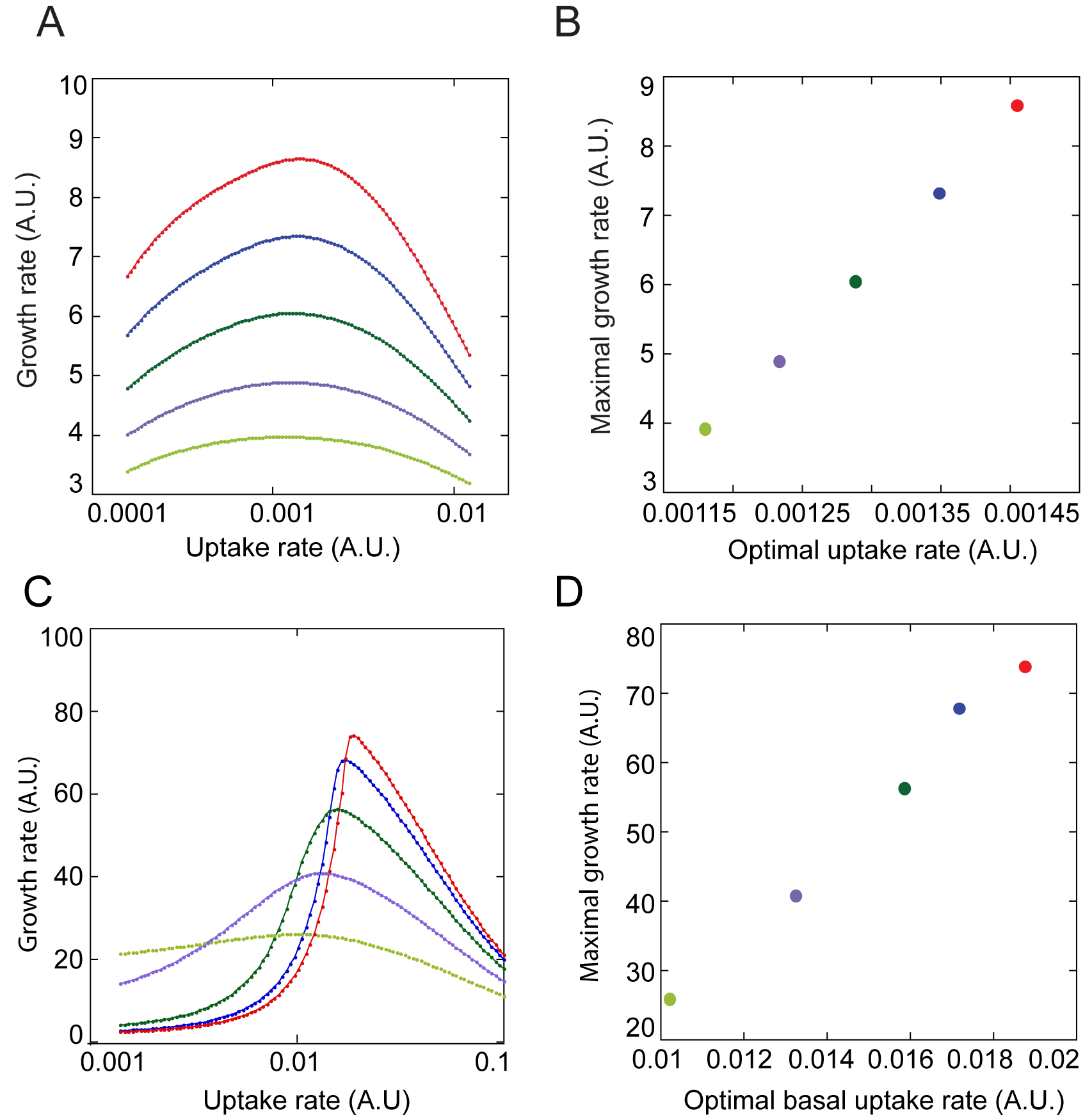

Supplement: S10 Fig — (A, B) Dependence of simulated cell growth on uptake rate for carbon sources of different metabolic efficiency for simulations performed as in Fig 5B but in absence of transporter induction by sugar-phosphate (A), and corresponding correlation between the optimal uptake rate and maximal growth rate for carbon sources of different metabolic efficiency (B). (C, D) Same as above but simulated for fixed maximal induction of transporter by sugar phosphate. See S1 Text for details. The underlying data can be found in S1 Data. (TIF) [file pbio.2000074.s010.tif]

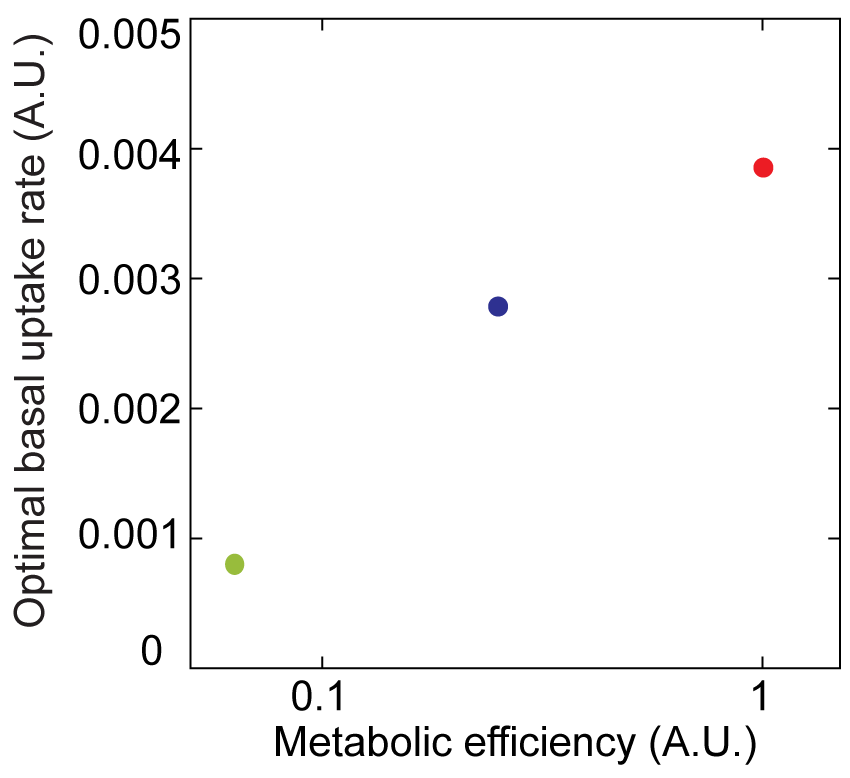

Supplement: S11 Fig — Correlation between the relative metabolic efficiency of the carbon source and its optimal basal uptake rate for simulated growth on three carbon sources. The underlying data can be found in S1 Data. (TIF) [file pbio.2000074.s011.tif]
