# Supplementary material for: Sugar Influx Sensing by the Phosphotransferase System of Escherichia coli
Source: PLoS Biol. 2016 Aug 24;14(8):e2000074. doi: 10.1371/journal.pbio.2000074 (PMC4996493; doi:10.1371/journal.pbio.2000074)
Supplement: S1 Table — (DOCX) [file pbio.2000074.s012.docx]

**S1 Table. FRET mapping of stimulus-dependent interactions between PTS proteins**

| CFP  YFP | EI | Hpr | EIIA^Glc^ | EIIBC^Glc^ | EIIAB^Man^ | EIIC^Man^ |
| --- | --- | --- | --- | --- | --- | --- |
| EI | -^a^ | - | - | - | - | - |
| Hpr | - | - | - | - | - | - |
| EIIA^Glc^ | - | - | - | - | - | - |
| EIICB^Glc^ | - | - | +^b^ | +^c^ | - | - |
| EIICBA^Nag^ | + | - | - | - | - | - |
| EIIAB^Man^ | - | - | - | - | - | - |
| EIIC^Man^ | - | - | - | - | + | - |
| EIICB^Mal^ | - | - | + | - | - | - |
| EIICBA^Mtl^ | - | - | - | N.D^d^ | N.D | N.D |
| EIICB^Tre^ | - | - | - | N.D | N.D | N.D |
| GalP | N.D | N.D | + | N.D | N.D | N.D |
| LacY | N.D | N.D | - | N.D | N.D | N.D |
| FucP | N.D | N.D | - | N.D | N.D | N.D |
| XylE | N.D | N.D | - | N.D | N.D | N.D |
| MglA | N.D | N.D | + | N.D | N.D | N.D |
| MalK | N.D | N.D | + | N.D | N.D | N.D |
| RbsA | N.D | N.D | + | N.D | N.D | N.D |
| UgpC | N.D | N.D | - | N.D | N.D | N.D |
| AraG | N.D | N.D | - | N.D | N.D | N.D |
| AlsA | N.D | N.D | - | N.D | N.D | N.D |

^a^Negative interaction (apparent FRET efficiency ≤0.5%)

^b^Positive interaction (apparent FRET efficiency >0.5%)

^c^Interaction observed for truncated EIIC^glc^-CFP – EIIC^glc^-YFP

^d^N.D – Not determined
