# Supplementary material for: Sugar Influx Sensing by the Phosphotransferase System of Escherichia coli
Source: PLoS Biol. 2016 Aug 24;14(8):e2000074. doi: 10.1371/journal.pbio.2000074 (PMC4996493; doi:10.1371/journal.pbio.2000074)
Supplement: S2 Table — (DOCX) [file pbio.2000074.s013.docx]

**S2 Table. List of metabolites tested for PTS response**

| **Compound (1mM)** | **PTS response** | **Normalized Response**  **(To 100 μM glucose)** |
| --- | --- | --- |
| 2-ketobutyrate | No | - |
| Alpha-ketoglutaric acid | No | - |
| Citric acid | No | - |
| Dihydoxyacetone | No | - |
| Dihydroxyacetone phosphate (DHAP) | No | - |
| fumarate | No | - |
| Gluconate | No | - |
| Glucose-6-phosphate | No | - |
| Glutaric acid | No | - |
| Glyceraldehyde-3-phosphate (GAP) | No | - |
| Glycerol | Yes | 0.82 ± 0.01 |
| Malate | No | - |
| Oxaloacetate | Yes | 0.28 ±0.01 |
| Phosphoenol pyruvate (PEP) | No | - |
| Pyruvate | Yes | 0.30 ± 0.01 |
| Rhamnose | No | - |
| Serine | Yes | 0.31± 0.01 |
| Shikimic acid | No | - |
| Succinate | No | - |
