# Supplementary material for: Sugar Influx Sensing by the Phosphotransferase System of Escherichia coli
Source: PLoS Biol. 2016 Aug 24;14(8):e2000074. doi: 10.1371/journal.pbio.2000074 (PMC4996493; doi:10.1371/journal.pbio.2000074)
Supplement: S3 Table — (DOCX) [file pbio.2000074.s014.docx]

**S3 Table. Lists of plasmids and strains used this study**

| **Plasmids** | **Relevant genotype** | **Induction** | **Source** |
| --- | --- | --- | --- |
| pBAD33 | Arabinose regulation (P_BAD_), pACYC ori, Cm^R^ | - | [1] |
| pTrc99a | IPTG regulation (Ptrc), pBR ori,Amp^R^ | - | [2] |
| pVS88 | cheZ-ecfp / cheY-eyfp, pTrc99a derivative | 50 μM IPTG | [3] |
| pVS344 | ptsI-eyfp, pTrc99a derivative | 40 μM IPTG | [4] |
| pVS346 | ptsG-eyfp ,pTrc99a derivative | 25 μM IPTG | [4] |
| pVS348 | ptsH-eyfp, pTrc99a derivative | 10 μM IPTG | [4] |
| pVS350 | ptsG-ecfp, pBAD33 derivative | 0.001% Ara | This work |
| pVS351 | ptsI-cfp, pBAD33 derivative | 0.01 % Ara | This work |
| pVS353 | crr-cfp, pBAD33 derivative | 0.001% Ara | This work |
| pVS355 | ptsH-ecfp, pBAD33 derivative | 10 μM IPTG |  |
| pVS445 | ptsG(EIIC)-eYFP, pTrc99a derivative | 40 μM IPTG | This work |
| pVS447 | manY-eyfp, pTrc99a derivative | 50 μM IPTG | This work |
| pVS448 | mtlA-eyfp, pTrc99a derivative | 30 μM IPTG | This work |
| pVS457 | lacY-eyfp, pTrc99a derivative | 40 μM IPTG | This work |
| pVS464 | ptsG(EIIC)-eCFP, pBAD33 derivative | 0.005 % Ara | This work |
| pVS1092 | Tar expression plasmid | 2 μM salicylate | [5] |
| pVS1314 | ugpC-eyfp, pTrc99a derivative | 40 μM IPTG | This work |
| pVS1315 | treB-eyfp, pTrc99a derivative | 25 μM IPTG | This work |
| pVS1317 | xylE-eyfp, pTrc99a derivative | 40 μM IPTG | This work |
| pVS1318 | malK-eyfp, pTrc99a derivative | 40 μM IPTG | This work |
| pVS1320 | araG-eyfp, pTrc99a derivative | 25 μM IPTG | This work |
| pVS1328 | alsA-eyfp, pTrc99a derivative | 20 μM IPTG | This work |
| pVS1330 | xylG-eyfp, pTrc99a derivative | 40 μM IPTG | This work |
| pVS1331 | fucP-eyfp,, pTrc99a derivative | 40 μM IPTG | This work |
| pVS1428 | crr-eyfp, pTrc99a derivative | 25 μM IPTG | [4] |
| pVS1435 | rbsA-eyfp, pTrc99a derivative | 40 μM IPTG | This work |
| pVS1436 | nagE-eyfp, pTrc99a derivative | 40 μM IPTG | This work |
| pVS1450 | manX-eyfp, pTrc99a derivative | 25 μM IPTG | This work |
| pVS1453 | galP-eyfp, pTrc99a derivative | 30 μM IPTG | This work |
| pVS1454 | mglA-eyfp, pTrc99a derivative | 35 μM IPTG | This work |
| pVS1455 | malX-eyfp, pTrc99a derivative | 35 μM IPTG | This work |
| pVS1465 | manX-ecfp,pBAD33 derivative | 0.001 % Ara | This work |
| pVS1467 | manY-ecfp,pBAD33 derivative | 0.001 % Ara | This work |
|  |  |  |  |
| **Strains** |  |  |  |
| LJ110 | W3110 Fnr^+^ (wild type) | – | [6] |
| RS1 | mglA-yfp and crr-cfp genomic fusion | – | This work |
| RS14 | LJ110 Δ *glpK*:: Kan |  | This work |
| RS4 | LJ110 Δ *trg* Δ *mglB* ::kan | - | This work |
| UU2612 | RP437 Δ(*tsr,tar,tap,trg,aer*) | - | [7] |
| JW0715 | BW25113 Δ*sucA*::Kan | - | [8] |
|  |  |  |  |

References

1. Guzman LM, Belin D, Carson MJ, Beckwith J. Tight regulation, modulation, and high-level expression by vectors containing the arabinose PBAD promoter. *J Bacteriol*. 1995;177(14):4121-30. PubMed PMID: 7608087; PubMed Central PMCID: PMCPMC177145.

2. Amann E, Ochs B, Abel KJ. Tightly regulated tac promoter vectors useful for the expression of unfused and fused proteins in *Escherichia coli*. *Gene*. 1988;69(2):301-15. PubMed PMID: 3069586.

3. Sourjik V. Receptor clustering and signal processing in E. coli chemotaxis. *Trends Microbiol*. 2004;12(12):569-76. doi: 10.1016/j.tim.2004.10.003. PubMed PMID: 15539117.

4. Neumann S, Grosse K, Sourjik V. Chemotactic signaling via carbohydrate phosphotransferase systems in *Escherichia coli*. *Proc Natl Acad Sci* USA. 2012;109(30):12159-64. doi: 10.1073/pnas.1205307109. PubMed PMID: 22778402; PubMed Central PMCID: PMC3409764.

5. Yang Y, A MP, Hofler C, Poschet G, Wirtz M, Hell R, et al. Relation between chemotaxis and consumption of amino acids in bacteria. *Mol Microbiol*. 2015;96(6):1272-82. doi: 10.1111/mmi.13006. PubMed PMID: 25807888.

6. Zeppenfeld T, Larisch C, Lengeler JW, Jahreis K. Glucose transporter mutants of Escherichia coli K-12 with changes in substrate recognition of IICB(Glc) and induction behavior of the ptsG gene. *J Bacteriol*. 2000;182(16):4443-52. PubMed PMID: 10913077; PubMed Central PMCID: PMCPMC94615.

7. Zhou Q, Ames P, Parkinson JS. Biphasic control logic of HAMP domain signalling in the *Escherichia coli* serine chemoreceptor. *Mol Microbiol*. 2011;80(3):596-611. doi: 10.1111/j.1365-2958.2011.07577.x. PubMed PMID: 21306449; PubMed Central PMCID: PMCPMC3095108.

8. Baba T, Ara T, Hasegawa M, Takai Y, Okumura Y, Baba M, et al. Construction of *Escherichia coli* K-12 in-frame, single-gene knockout mutants: the Keio collection. *Mol Syst Biol.* 2006;2:2006 0008. doi: 10.1038/msb4100050. PubMed PMID: 16738554; PubMed Central PMCID: PMCPMC1681482.
