# Supplementary material for: Sugar Influx Sensing by the Phosphotransferase System of Escherichia coli
Source: PLoS Biol. 2016 Aug 24;14(8):e2000074. doi: 10.1371/journal.pbio.2000074 (PMC4996493; doi:10.1371/journal.pbio.2000074)
Supplement: S1 Text — (PDF) [file pbio.2000074.s015.pdf]

S1 Text. Mathematical model of PTS-mediated regulation

The schematic reaction scheme for the PTS sugar utilization system assumed in our model is as follows:

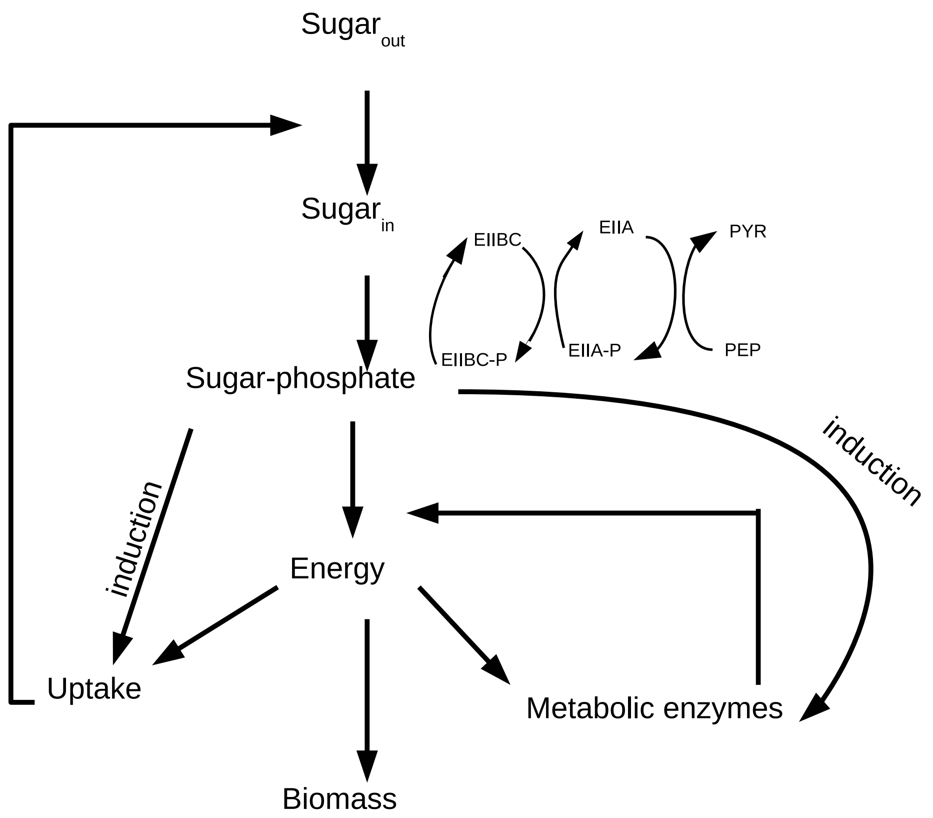

The uptake of sugar depends on the total number of transporters as well as the binding affinity of sugar to the transporter. The sugar is further phosphorylated by the transporter to capture the sugar molecule inside the cytoplasm. We assume that the binding of sugar to the transporter happens at much faster time scale than the phosphorylation events. So the binding-unbinding events reach steady state before the phosphotransfer reactions. The sugar-phosphate induces transcription of its specific metabolic enzymes and transporters. Energy is consumed in biomass production as well as uptake of the sugar and synthesis of proteins. Here we considered three cases, with (i) transcriptional induction of fixed maximum fold relative to the basal rate of uptake; (ii) fixed maximal transcriptional induction independent of the basal uptake; and (iii) no transcriptional induction. Notably, we assume that metabolic enzymes are induced by their substrates in all cases, with a maximal induction being equal for all sugars.

The set of reactions for the above-mentioned scheme is given by

Uptake of sugar

$$\overset{x_1}{sugar_{out}} \underset{k_{-u}}{\overset{k_U}{\rightleftharpoons}} \overset{x_2}{sugar_n}, k_U = k_u\left(\overset{x_9}{EIIBC} + \overset{x_8}{EIIBC_P}\right)\frac{sugar_{out}}{K_s + sugar_{out}}$$

Transfer of phosphate to EIIA from PEP

$$\overset{x_3}{EIIA} + \overset{x_5}{PEP} \underset{k_{-t}}{\overset{k_t}{\rightleftharpoons}} \overset{x_4}{EIIA_P} + \overset{x_6}{PYR}$$

Conversion of sugar to sugar-phosphate

$$\overset{x_2}{sugar_n} + \overset{x_8}{EIIBC_P} \underset{k_{-c}}{\overset{k_C}{\rightleftharpoons}} \overset{x_9}{EIIBC} + \overset{x_7}{sugarphosphate}$$

Transfer of phosphate to EIIBC from EIIA-P

$$\overset{x_9}{EIIBC} + \overset{x_4}{EIIA_P} \underset{k_{-p}}{\overset{k_p}{\rightleftharpoons}} \overset{x_8}{EIIBC_P} + \overset{x_3}{EIIA}$$

Production of energy from sugar-phosphate by metabolic protein$m_p$and consumption of energy in uptake and metabolic enzyme production

$$sugarphosphate \overset{\alpha m_p}{\longrightarrow} E \overset{\gamma_{biomass}}{\longrightarrow} M, E \overset{\gamma_{uptake} EIIBC_{tot}}{\longrightarrow} \phi, E \overset{\gamma_{enzyme} m_p}{\longrightarrow} \phi$$

Induction of the transporter EIIBC by sugar-phosphate

$$G \overset{\beta}{\longrightarrow} \overset{x_9}{EIIBC} \overset{\gamma}{\longrightarrow} \phi, \beta = \beta_0 + \beta_{induced}\frac{sugarphosphate}{K + sugarphosphate}$$

For the case of no induction of the transporter, $\beta_{induced} = 0$

Induction of metabolic enzyme by sugar-phosphate

$$G \overset{\beta\prime}{\longrightarrow} \overset{x_{10}}{m_p} \overset{\gamma}{\longrightarrow} \phi, \beta' = \beta_{e0} + \beta_{einduced}\frac{sugarphosphate}{K + sugarphosphate}$$

The corresponding equations for the dynamics of the reactants (based on mass action kinetics) are

Sugar uptake

$$\frac{dx_1}{dt} = -k_u(x_8 + x_9)\frac{x_1}{K_S + x_1} + k_{-u}x_2$$

Reaction for intracellular sugar

$$\frac{dx_2}{dt} = k_u(x_8 + x_9)\frac{x_1}{K_S + x_1} - k_{-u}x_2 - k_c x_8 x_2 + k_{-c}x_9 x_7$$

Reaction for EIIA

$$\frac{dx_3}{dt} = -k_t x_3 x_5 + k_{-t}x_4 x_6 + k_p x_9 x_4 - k_{-p}x_8 x_3$$

Reaction for EIIA-P

$$\frac{dx_4}{dt} = k_t x_3 x_5 - k_{-t}x_4 x_6 - k_p x_9 x_4 + k_{-p}x_8 x_3$$

Reaction for PEP

$$\frac{dx_5}{dt} = \beta_{PEP} - k_t x_3 x_5 + k_{-t}x_4 x_6 - \gamma_{PEP}x_5$$

Reaction for PYR

$$\frac{dx_6}{dt} = \beta_{PYR} + k_t x_3 x_5 - k_{-t}x_4 x_6 - \gamma_{PYR}x_6$$

Reaction for sugar-phosphate

$$\frac{dx_7}{dt} = k_c x_8 x_2 - k_{-c}x_9 x_7 - \alpha x_{10}x_7$$

Reaction of EIIB-P

$$\frac{dx_8}{dt} = k_p x_4 x_9 + k_{-p}x_3 x_8 - k_c x_8 x_2 + k_{-c}x_9 x_7 - \gamma x_8$$

Reaction for EIIBC

$$\frac{dx_9}{dt} = \beta_0 + \beta_{induced}\frac{x_7}{K + x_7} - \gamma x_9 - k_p x_4 x_9 + k_{-p}x_3 x_8 + k_c x_8 x_2 - k_{-c}x_9 x_7$$

Induction of metabolic enzymes

$$\frac{dx_{10}}{dt} = \beta_{e0} + \beta_{einduced}\frac{x_7}{K + x_7} - \gamma x_{10}$$

Production and consumption of energy

$$\frac{dE}{dt} = \alpha_0 + \alpha x_{10}x_7 - \gamma_{biomass}E - \gamma_{uptake}(x_8 + x_9)E - \gamma_{enzyme}x_{10}E$$

Biomass production

$$\frac{dM}{dt} = \gamma_{biomass}E$$

Growth of the population of cells

$$G = e^{\lambda M}$$

The values of the reaction constants:

Phosphotransfer reaction:

$$K_s = 5mM;$$
$$k_u = k_c = k_t = k_p = 0.001\mu M^{-1}ms^{-1} \sim 100\mu M^{-1}min^{-1}$$
$$k_{-u} = 10^{-7}\mu M^{-1}ms^{-1}; k_{-c} = 0; k_{-t} = k_{-p} = 0.00001\mu M^{-1}ms^{-1} \sim 1\mu M^{-1}min^{-1}$$

Induction and dilution of enzymes:

for the case of fixed maximum fold induction (i)

$$k_u = k_c = k_t = k_p = 0.001\mu M^{-1}ms^{-1} \sim 100\mu M^{-1}min^{-1}$$
$$\beta_{e0} = 10^{-6}\mu Mms^{-1} \sim 0.1\mu Mmin^{-1}; \beta_{einduced} = 50\beta_{e0} \sim 5\mu Mmin^{-1}; \gamma = 10^{-7}ms^{-1} \sim 0.01min^{-1};$$
$$K = 1000\mu M$$
$$\beta_0 = 1.4\times10^{-7} - 1.08\times10^{-5}\mu Mms^{-1}; \beta_{induced} = 50\beta_0;$$

for the case of fixed maximum induction (ii)

$$k_u = k_c = k_t = k_p = 0.001\mu M^{-1}ms^{-1} \sim 100\mu M^{-1}min^{-1}$$
$$\beta_{e0} = 10^{-6}\mu Mms^{-1} \sim 0.1\mu Mmin^{-1}; \beta_{einduced} = 50\beta_{e0} \sim 5\mu Mmin^{-1}; \gamma = 10^{-7}ms^{-1} \sim 0.01min^{-1};$$
$$K = 1000\mu M$$
$$\beta_0 = 1.4\times10^{-7} - 1.08\times10^{-5}\mu Mms^{-1}; \beta_{induced} = 0.5\times10^{-4}\mu Mms^{-1};$$

for the case of no induction (iii)

$$k_u = k_c = k_t = k_p = 0.01\mu M^{-1}ms^{-1} \sim 1000\mu M^{-1}min^{-1}$$
$$\beta_{e0} = 10^{-6}\mu Mms^{-1} \sim 0.1\mu Mmin^{-1}; \beta_{einduced} = 50\beta_{e0} \sim 5\mu Mmin^{-1}; \gamma = 10^{-7}ms^{-1} \sim 0.01min^{-1};$$
$$K = 1000\mu M$$
$$\beta_0 = 5.1\times10^{-9} - 4.08\times10^{-7}\mu Mms^{-1}; \beta_{induced} = 0;$$

Production and consumption of energy

$$\alpha_0 = 0.001ms^{-1}; \gamma_{biomass} = 10^{-7}\mu M^{-1}ms^{-1} \sim 0.01\mu M^{-1}min^{-1}$$
$$\gamma_{uptake} = \gamma_{enzyme} = 0.2\times10^{-7}\mu M^{-1}ms^{-1} \sim 0.002\mu M^{-1}min^{-1};$$
$$\lambda = 10^{-4}\mu M^{-1}; \beta_{PEP} = \beta_{PYR} = 0.02\mu Mms^{-1}; \gamma_{PEP} = \gamma_{PYR} = 10^{-5}ms^{-1}$$

Initial Concentrations:

$$sugar = 200mM; EIIA_{tot} = 40\mu M; PEP = 3000\mu M; PYR = 1000\mu M; ATP_0 = 500\mu M$$

The equations are simulated for $10^7 ms \sim 100min$ for 5 different values of the nutritional value$\alpha$with three fold differences and 100 different values of the uptake rate for each value ofα. The biomass

produced is calculated in these 100 min. The uptake rate which delivers the highest growth-rate is defined as the optimal uptake rate (transporter number multiplied by the uptake rate constant) for a particular nutritional value of a sugar. We used CVode [1] interfaced with MATLAB to solve the ODEs.

Solution of the simple model

The simple cost benefit model for the utilization and uptake of the sugar can be written as

$$\frac{dE}{dt} = \alpha X^2 - \beta X E \qquad (1)$$

where $\alpha$and βrepresent the nutritional value and the cost of uptake respectively. X indicates the uptake rate.

Solving the equation for $E(t)$

$$\beta E(t) = \alpha X - (\alpha X - \beta E_0)e^{\frac{-t}{\beta X}} \qquad (2)$$

to obtain the optimal uptake rate, $\frac{dE(t)}{dX} = 0$, by taking derivative of equation (2) we obtain

$$\beta \frac{dE(t)}{dX} = e^{\frac{-t}{\beta X}}\left(1 + \frac{t}{\beta X} - \frac{E_0 t}{\alpha X^2}\right) - \alpha = 0 for X = X_{opt} \qquad (3)$$

Taking derivative of equation (3) with respect to α,

$$\frac{dX_{opt}}{d\alpha} = \frac{\frac{t}{\beta X_{opt}^2} e^{\frac{-t}{\beta X_{opt}}} + \frac{E_0 t}{\alpha^2 X_{opt}^2}}{\frac{t}{\alpha X_{opt}^3}(E_s - 2E_0)} \qquad (4)$$

Equation (4) implies that $\frac{dX_{opt}}{d\alpha} > 0$ when $E_s > 2E_0$ suggesting that optimal uptake rate would increase with the nutritional value as long as energy produced during sugar uptake is much higher than the energy produced during starvation.

The optimal uptake rate corresponds to the basal uptake rate at which the biomass produced within a certain amount of time is maximal. If the basal uptake rate is below the optimum, it limits cell growth (biomass production). As the basal uptake rate is increased, the sugar-phosphate accumulates inside the cell, inducing the uptake. Consequently, high basal uptake rate leads to high induced uptake rate, ultimately resulting in the energy consumption in uptake being disproportionally high compared to the energy production by metabolism of the sugar. At an intermediate level of the basal uptake, the energy production and energetic cost are balanced, giving rise to an optimal basal uptake rate. In this scenario,

for a sugar with a low metabolic efficiency the induction of uptake occurs at low basal uptake rate, since the sugar-phosphate accumulates due to low rate of its metabolism. In contrast, the same basal uptake rate would not lead to uptake induction for a sugar with high metabolic efficiency, because the corresponding sugar-phosphate would not accumulate due to its faster metabolism. As a consequence, the induction will occur at higher basal uptake rate, thus leading to higher optimal basal uptake rates for sugars with higher metabolic efficiency.

Model for sugar utilization in a mixture of PTS sugars

When more than one PTS sugar is available in the environment, *E.coli* can utilize both sugars simultaneously. Inside the cytoplasm, the sugars are phosphorylated by the same phosphate source. The model is first constructed for a mixture of two sugars. We assume that the sugars can only be taken up by their specific transporters and the phosphate transfer occurs from the phosphate source EIIA-P to both the sugars. The nutritional values are only different for the two sugars. The rates of phosphotransfer reactions are assumed to be the same. The transporter and the metabolic enzymes are induced by the corresponding sugar-phosphate. The simulations are performed for 20 different values of the basal uptake rate for each of the sugars. The two uptake rates for which the growth rate is maximum indicate the optimal basal uptake rates for the two sugars in mixture. We carried out the simulation for five different mixture of two sugars by changing the nutritional value of one of the sugars, keeping the other sugar fixed and the biomass produced was calculated within 100 min with a periodic (every 20 min) resupply of sugars.

Uptake of sugar[1]

$$\overset{x_1}{sugar^1_{out}} \underset{k_{-u}}{\overset{k_U}{\rightleftharpoons}} \overset{x_2}{sugar^1_n}, k_U = k_u\left(\overset{x_9}{EIIBC^1} + \overset{x_8}{EIIBC^1_P}\right)\frac{sugar^1_{out}}{K_s + sugar^1_{out}}$$

Transfer of phosphate to EIIA from PEP

$$\overset{x_3}{EIIA} + \overset{x_5}{PEP} \underset{k_{-t}}{\overset{k_t}{\rightleftharpoons}} \overset{x_4}{EIIA_P} + \overset{x_6}{PYR}$$

Conversion of sugar1 to sugar-phosphate[1]

$$\overset{x_2}{sugar^1_n} + \overset{x_8}{EIIBC^1_P} \underset{k_{-c}}{\overset{k_c}{\rightleftharpoons}} \overset{x_9}{EIIBC^1} + \overset{x_7}{sugarP^1}$$

Transfer of phosphate to EIIBC[1] from EIIA-P

$$\overset{x_9}{EIIBC^1} + \overset{x_4}{EIIA_P} \underset{k_{-p}}{\overset{k_p}{\rightleftharpoons}} \overset{x_8}{EIIBC_P^1} + \overset{x_3}{EIIA}$$

Uptake of sugar[2]

$$\overset{x_{13}}{sugar_{out}^2} \underset{k_{-u}}{\overset{k_U}{\rightleftharpoons}} \overset{x_{14}}{sugar_n^2}, k_U = k_u \left(\overset{x_{16}}{EIIBC^2} + \overset{x_{15}}{EIIBC_P^2}\right) \frac{sugar_{out}^2}{K_s + sugar_{out}^2}$$

Conversion of sugar[2] to sugar-phosphate[2]

$$\overset{x_{14}}{sugar_n^2} + \overset{x_{15}}{EIIB\ C_P^2} \underset{k_{-c}}{\overset{k_c}{\rightleftharpoons}} \overset{x_{16}}{EIIBC^2} + \overset{x_{17}}{sugar\ P}^{\ 2}$$

Transfer of phosphate to EIIBC[2] from EIIA-P

$$\overset{x_{16}}{EIIBC^2} + \overset{x_4}{EIIA_P} \underset{k_{-p}}{\overset{k_p}{\rightleftharpoons}} \overset{x_{15}}{EIIBC_P^2} + \overset{x_3}{EIIA}$$

Production of energy from sugar-P[1,2] by metabolic protein$m_p^{1,2}$and consumption of energy in uptake and metabolic enzymes production

$$sugarP^1 + sugarP^2 \overset{\alpha_1 m_p^1 + \alpha_2 m_p^2}{\longrightarrow} E \overset{\gamma_{biomass}}{\longrightarrow} M, E \overset{\gamma_{uptake} EIIBC_{tot}^{1+2}}{\longrightarrow} \phi, E \overset{\gamma_{enzyme} m_p^{1+2}}{\longrightarrow} \phi$$

Induction of the transporter EIIBC1,2 by sugar-phosphate[1,2]

$$G \overset{\beta^{1,2}}{\longrightarrow} \overset{x_9}{EIIBC^{1,2}} \overset{\gamma}{\longrightarrow} \phi, \beta^{1,2} = \beta_0^{1,2} + \beta_{induced}^{1,2} \frac{sugarP^{1,2}}{K + sugarP^{1,2}}$$

Induction of metabolic enzyme by sugar-phosphate[1,2]

$$G \overset{\beta'}{\longrightarrow} \overset{x_{10}, x_{18}}{m}{}_p^{1,2} \overset{\gamma}{\longrightarrow} \phi, \beta' = \beta_{e0} + \beta_{einduced} \frac{sugarP^{1,2}}{K + sugarP^{1,2}}$$

We assume following values of the reaction constants:

Phosphotransfer reactions

$$K_s = 5mM;$$
$$k_u = k_c = k_t = k_p = 0.001\mu M^{-1} ms^{-1} \sim 100\mu M^{-1} min^{-1}$$
$$k_{-u} = k_{-c} = k_{-t} = k_{-p} = 0.00001\mu M^{-1} ms^{-1} \sim 1\mu M^{-1} min^{-1}$$

Induction and dilution of enzymes

$$\beta_{e0} = 10^{-6}\mu M ms^{-1} \sim 0.1\mu M min^{-1}; \beta_{einded} = 50\beta_{e0} \sim 5\mu M min^{-1}; \gamma = 10^{-7} ms^{-1} \sim 0.01 min^{-1}; K = 1000\mu M$$

Production and consumption of energy

$$\alpha_0 = 0.001 ms^{-1}; \gamma_{biomass} = 10^{-7}\mu M^{-1} ms^{-1} \sim 0.01\mu M^{-1} min^{-1}$$
$$\gamma_{uptakt} = \gamma_{enzyme} = 0.2\times10^{-7}\mu M^{-1} ms^{-1} \sim 0.002\mu M^{-1} min^{-1};$$
$$\lambda = 10^{-4}\mu M^{-1}; \beta_{PEP} = \beta_{PYR} = 0.02\mu M ms^{-1}; \gamma_{PEP} = \gamma_{PYR} = 10^{-5} ms^{-1}$$

Initial concentrations

$$sugar^1 = 10mM; sugar^2 = 10mM; EIIA_{tot} = 40\mu M; PEP = 3000\mu M; PYR = 1000\mu M; ATP_0 = 500\mu M$$

Similarly, one more sugar is added in the model to construct a mixture of 3 sugars. The three sugars differ by four fold in the nutritional values. The equations are simulated by varying the basal uptake rates of the three sugars and the three basal uptake rates are obtained which display maximum growth rate. In this case, the three sugars compete with each other for transport inside cell, since all transporters utilize the same phosphate source (PEP). However, the transcriptional activation of the transporter and the metabolic enzymes for a particular sugar is specifically induced by the corresponding sugar-phosphate. As a result, the sugar for which the metabolic efficiency is highest has the highest optimal basal uptake rate in mixture.

Reference

[1] Vanlier J, Tiemann CA, Hilbers PA, van Riel NA. An integrated strategy for prediction uncertainty analysis. Bioinformatics. 2012;28(8):1130–5
